# Specific Human and Candida Cellular Interactions Lead to Controlled or Persistent Infection Outcomes during Granuloma-Like Formation

**DOI:** 10.1128/IAI.00807-16

**Published:** 2016-12-29

**Authors:** Barbara Misme-Aucouturier, Marjorie Albassier, Nidia Alvarez-Rueda, Patrice Le Pape

**Affiliations:** Département de Parasitologie et de Mycologie Médicale, Université de Nantes, Nantes Atlantique Universités, EA1155-IICiMed, Institut de Recherche en Santé 2, Nantes, Pays de Loire, France; University of Cincinnati

**Keywords:** Candida spp., granulomatous immune response, host-pathogen interactions, persistence

## Abstract

A delayed type of multicellular process could be crucial during chronic candidiasis in determining the course of infection. This reaction, consisting of organized immune cells surrounding the pathogen, initiates an inflammatory response to avoid fungal dissemination. The goal of the present study was to examine, at an *in vitro* cellular scale, Candida and human immune cell interaction dynamics during a long-term period. By challenging human peripheral blood immune cells from 10 healthy donors with 32 Candida albicans and non-*albicans* (C. glabrata, C. tropicalis, C. parapsilosis, C. dubliniensis, C. lusitaniae, C. krusei, and C. kefyr) clinical isolates, we showed that Candida spp. induced the formation of granuloma-like structures within 6 days after challenge, but their sizes and the respective fungal burdens differed according to the Candida species. These two parameters are positively correlated. Phenotypic characteristics, such as hypha formation and higher axenic growth rate, seem to contribute to yeast persistence within granuloma-like structures. We showed an interindividual variability of the human response against Candida spp. Higher proportions of neutrophils and elevated CD4^+^/CD8^+^ T cell ratios during the first days after challenge were correlated with early production of gamma interferon (IFN-γ) and associated with controlled infection. In contrast, the persistence of Candida could result from upregulation of proinflammatory cytokines such as interleukin-6 (IL-6), IFN-γ, and tumor necrosis factor alpha (TNF-α) and a poor anti-inflammatory negative feedback (IL-10). Importantly, regulatory subsets of NK cells and CD4^lo^ CD8^hi^ doubly positive (DP) lymphocytes at late stage infiltrate granuloma-like structures and could correlate with the IL-10 and TNF-α production. These data offer a base frame to explain cellular events that guide infection control or fungal persistence.

## INTRODUCTION

Candidiasis still constitutes a global health threat. The annual invasive candidiasis incidence reported by international population-based studies has been estimated at 1.5 to 8 per 100,000 (1; http://www.gaffi.org). The global mortality rate of bloodstream infections (BSI) varies between 30 and 50% (2). Candida species have coevolved with humans, colonizing different body sites such as the gastrointestinal mucosa, genitourinary system, and skin microbiota (3, 4). In healthy individuals, complex relationships between some Candida species and the human immune system make the fungus a harmless commensal. Prominent features such as the yeast genetic background, the ability of some species to reversely switch from yeast to hyphae, and the quality of antifungal immune responses at different anatomical sites guide host-fungus interaction. With the continued progress in the understanding of innate immune sensing of Candida spp., it is now clear that a balanced host-fungus interaction is a condition for Candida commensalism (5–8). Candida infections occur in patients with specific risk factors, especially a dysfunction of the innate immune system such as neutropenia or a disruption of physical barriers, leading to the dissemination of Candida. The Candida species of major clinical importance are Candida albicans and other non-albicans Candida spp. (NACs) such as C. glabrata, C. tropicalis, C. parapsilosis, C. lusitaniae, C. krusei, and C. kefyr (9–11). A low frequency of infections by C. dubliniensis has also been described for neutropenic patients, but the mortality is higher than with other NACs (12). The spectrum of clinical manifestations of candidiasis includes cutaneous, mucosal, systemic, and disseminated candidiasis. Disseminated infections may be either acute or chronic depending on the onset of fungemia and organ dissemination.

Thanks to the recent publication of the revolutionary concept of a damage response framework (DRF) from the perspective of candidiasis, it has become clear that host and Candida interaction is a more complex outcome. It is now well established that infections can be raised not only because of host-mediated damage but also because of fungus-mediated damage or both (13). Remarkable advances in the understanding of the pathophysiology of Candida infections highlight that multiple cell populations are involved in the anti-Candida response. Furthermore, innate and adaptive immune requirements for human defense are specific and compartmentalized between mucosal and systemic infections (14, 15). It is well accepted that anti-Candida responses require the coordinated action of neutrophil and macrophage phagocytosis that can clear the fungus and further activate the release of proinflammatory cytokines, which protect from fungal dissemination. Dendritic cells, natural killer cells, and B and T lymphocytes (CD4 and CD8) also play central roles (16–21).

In the context of chronic disseminated candidiasis, the delayed multicellular inflammatory process could be particularly essential to manage fungal clearance, to protect the surrounding healthy tissue, and to prevent fungal persistence. Granulomas, which correspond to a focal area of granulomatous inflammatory reaction, are composed of blood-derived infected and uninfected macrophages, differentiated macrophages (epithelioid cells), and multinucleated giant cells, surrounded by a ring of T and B lymphocytes. Granuloma formation during Candida dissemination in humans is occasionally described, and its role in pathophysiology has not been well investigated. During invasive candidiasis, at-risk patients develop suppurative inflammation with rare granulomas lesions in different organs (22). Histopathologic descriptions of microabscesses and/or sparse granulomas have been reported for the liver, spleen, kidneys, and brain (23–27). Hepatic lesions are usually characterized by areas of central necrosis or fibrosis, surrounded by granulation tissue, macrophages, fibroblasts, and multinuclear giant cells (27). While these delayed multicellular reactions are clinically rare during candidiasis, they could play a role in controlling infection at local tissue levels. A better dissection of these reactions could allow the identification of fungal and human determinants of clearance or persistence.

Experimental animal models have yielded a tremendous amount of information about C. albicans pathogenesis. These clinically relevant models have been particularly useful to study systemic candidiasis. In these models, C. albicans dissemination causes extensive disease, mostly in the kidneys, that are also characterized by multiple compact immune infiltrates (28–30). *In vitro* studies describing the infection of peripheral blood mononuclear cells (PBMC) are complementary to animal models giving information about Candida pathogenesis in the context of human-like environments. The *in vitro* models have been particularly useful to study pathogenesis and virulence factors of Candida spp. (31–36). Human PBMC-related *in vitro* models have been validated for the study of Mycobacterium tuberculosis and Schistosoma mansoni granuloma-like structures. They recapitulate the complexity of host responses and mimic the microenvironment encountered by the pathogen within the human granuloma-like structures (37–39).

We have previously set up an *in vitro* model to recreate the multicellular interaction of human immune cells with C. albicans (40). In this study, we aimed to address the gap in knowledge about the relevance of these dense immune infiltrates by analyzing other clinical relevant Candida species. We then analyzed at a cellular level the dynamics of immune infiltrate formation over time by challenging peripheral mononuclear and polymorphonuclear human leukocytes from 10 healthy donors with 32 C. albicans and NAC clinical isolates. Morphometric analyses of cellular interactions in terms of size and granuloma-like structure number, fungal burden, immune cell dynamics, and cytokine profiles were carried out. The data should provide a baseline to explain cellular events conditioning infection control or fungal persistence outcomes.

## RESULTS

### Candida spp. induced the formation of dense immune infiltrates within 6 days after challenge.

Peripheral mononuclear and polymorphonuclear blood cells from 10 healthy immunocompetent subjects were challenged with 32 clinical isolates from eight Candida species. Cell cultures were maintained for up to 6 days postinfection, and the occurrence of immune infiltrates was quantified every 2 days by light microscopy observation.

**FIG 1:**
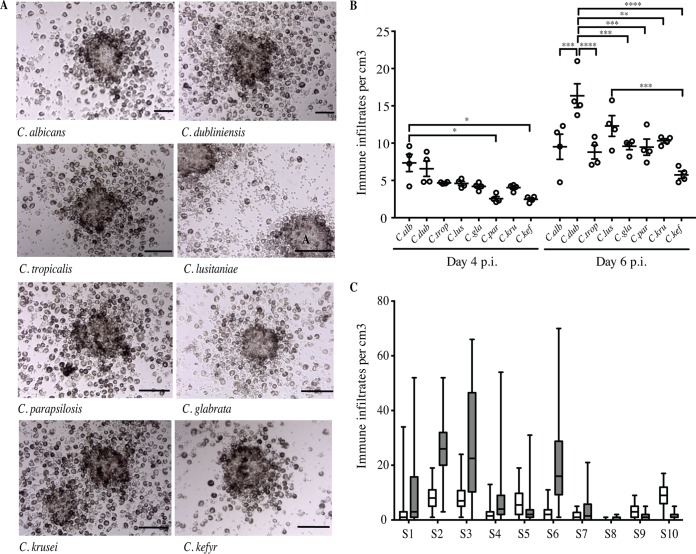
*In vitro* immune infiltrates induced by Candida spp. after infection of human immune cells from immunocompetent subjects. (A) Representative immune infiltrates observed under light microscopy 6 days after challenge of human mononuclear and polymorphonuclear peripheral blood cells with living yeasts from annotated Candida species (MOI, phagocyte-to-yeast ratio of 2,000:1). Bars represent 50 μm. No formation of immune infiltrates was observed in uninfected conditions for up to 6 days postinfection (p.i.). (B) Number of immune infiltrates per cubic centimeter on days 4 and 6 postinfection by the eight Candida species. Each dot represents the mean of the number of structures per cubic centimeter for one clinical isolate and for all studied subjects. Lines indicate SEM (*, *P* between 0.045 and 0.0360; **, *P* = 0.0017; ***, *P* between 0.0004 and 0.0002; ****, *P* < 0.0001; α = 0.05). *C.alb*, C. albicans; *C.dub*, C. dubliniensis; *C.trop*, C. tropicalis; *C.lus*, C. lusitaniae; *C.gla*, C. glabrata; *C. par*, C. parapsilosis; *C.kru*, C. krusei; *C.kef*, C. kefyr. (C) Box plots depict median, minimum, and maximum immune infiltrate numbers for each donor subject (S) on days 4 (white boxes) and 6 (gray boxes) postinfection (*n* = 80).

Both uninfected and infected conditions showed monolayers of cells 2 days postinfection. Cellular aggregation was visible 4 days after challenge. Between days 4 and 6, distinguishable multicellular and multilayered structures characteristic similar granuloma-like structures were identified under infection conditions. At that time, the 32 clinical isolates of Candida spp. were able to induce dense aggregates. Figure 1A shows representative compact immune infiltrates observed with each of the eight Candida species 6 days after challenge. On day 4 postinfection, the average number of these structures was between 2.5 (C. kefyr) and 7.4 (C. albicans) per cm^3^ (Fig. 1B). On day 6, the number of structures increased for all species, with no significant differences compared to those on day 4 (*P* = 0.05). The formation of these structures against Candida spp. also varied depending on the donor subject. Figure 1C shows that subjects S1, S2, S3, S4, S6, and S7 developed granuloma-like structures on day 4 after infection. The number of these structures increased between 2.7- and 10-fold on day 6 postinfection. In contrast, a proportion of subjects (S5, S8, S9, and S10) developed significantly fewer immune infiltrates during the 6 days of infection culture. Unchallenged cultures from the same subjects, used as negative controls, exhibited no specific aggregates within 6 days.

### The size of immune infiltrates structures induced by Candida spp. progressively increased over time.

**FIG 2:**
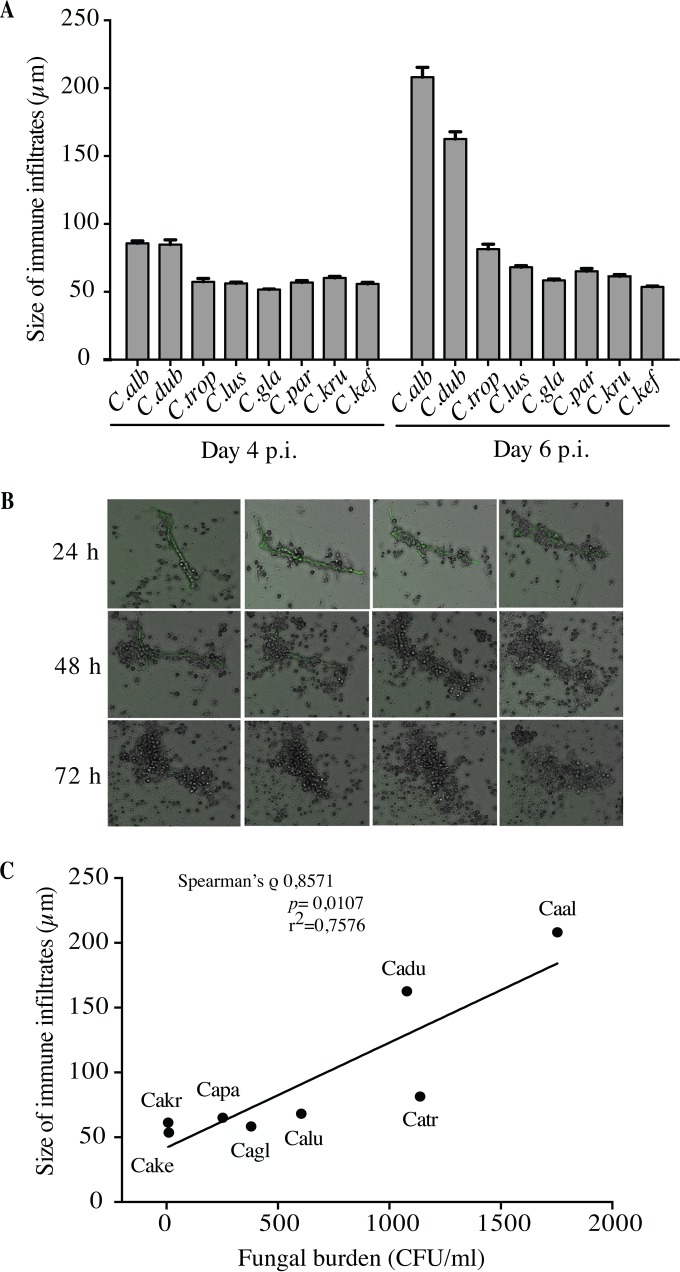
Dynamics of immune infiltrate size according to Candida species. (A) Human mononuclear and polymorphonuclear peripheral blood cells from donors were infected with Candida spp. for up to 6 days. The size of immune infiltrates was measured by light microscopy on days 4 and 6 postinfection. The data are presented as the mean + SEM of structure sizes from 10 subjects for 32 clinical isolates of Candida species 6 days postinfection. (B) Coculture analysis by time-lapse video imaging. Human peripheral blood mononuclear and polymorphonuclear cells were infected with C. albicans cells at an MOI of 2,000:1. Cell aggregation was followed for 72 h, and a capture was done every 10 min. Bars represent 50 μm. (C) Pairwise correlation of immune infiltrates size and fungal burden. Shown is a graphical representation of the fungal burden means (*x* axis) and immune infiltrate size (*y* axis) 6 days postinfection. The line indicates the slope.

The size of immune infiltrates was quantified under light microscopy during the incubation time. They were measured on days 4 and 6 postinfection, since multicellular interactions consistently showed cellular aggregation at these time points. The average sizes of the immune infiltrates induced by the 32 clinical isolates of eight Candida species after challenge of 10 subjects were similar when measured 4 days postinfection (mean size of 63 μm) (Fig. 2A). Two days later, the relative sizes of immune infiltrates were unchanged for C. tropicalis, C. lusitaniae, C. glabrata, C. parapsilosis, C. krusei, and C. kefyr (Fig. 2A). In contrast, for C. albicans and C. dubliniensis, the sizes of immune infiltrates were significantly higher than for the six other species (208 μm and 163 μm, respectively). At that time, light microscopic observation of these structures derived from C. albicans and C. dubliniensis showed the development of some spreading hyphae and pseudohyphae. As shown in Fig. 2B, differences in the magnitude of recruitment were generally observed when cell aggregation occurred around these filaments. Time-lapse imaging of C. albicans-green fluorescent protein (GFP) cocultures also showed the fusion of compact immune infiltrates formed around filaments, giving rise to larger structures (see Videos S1 and S2 in the supplemental material). Despite interindividual variability in the numbers of immune infiltrates generated by Candida spp., no significant difference was observed between subjects regarding the structure size.

### Size of immune infiltrates induced by Candida spp. was positively correlated with fungal burden.

We next explored whether immune infiltrates sizes were related to the fungal burden for all the clinical isolates of Candida spp. Overall, a significant positive correlation was observed between the average size of immune infiltrates and the fungal burden on day 6 postinfection, with a high Spearman's correlation coefficient (ρ = 0.8571; *P* = 0.0107) (Fig. 2C). Therefore, C. albicans and C. dubliniensis, which induced large immune infiltrates, were associated with a high fungal burden, reflecting an *in vitro* uncontrolled infection. Conversely, the range of immune infiltrate sizes observed with the other species was directly correlated with a lower fungal burden.

### The dynamics of the fungal burden within immune infiltrates were different between Candida species.

**FIG 3:**
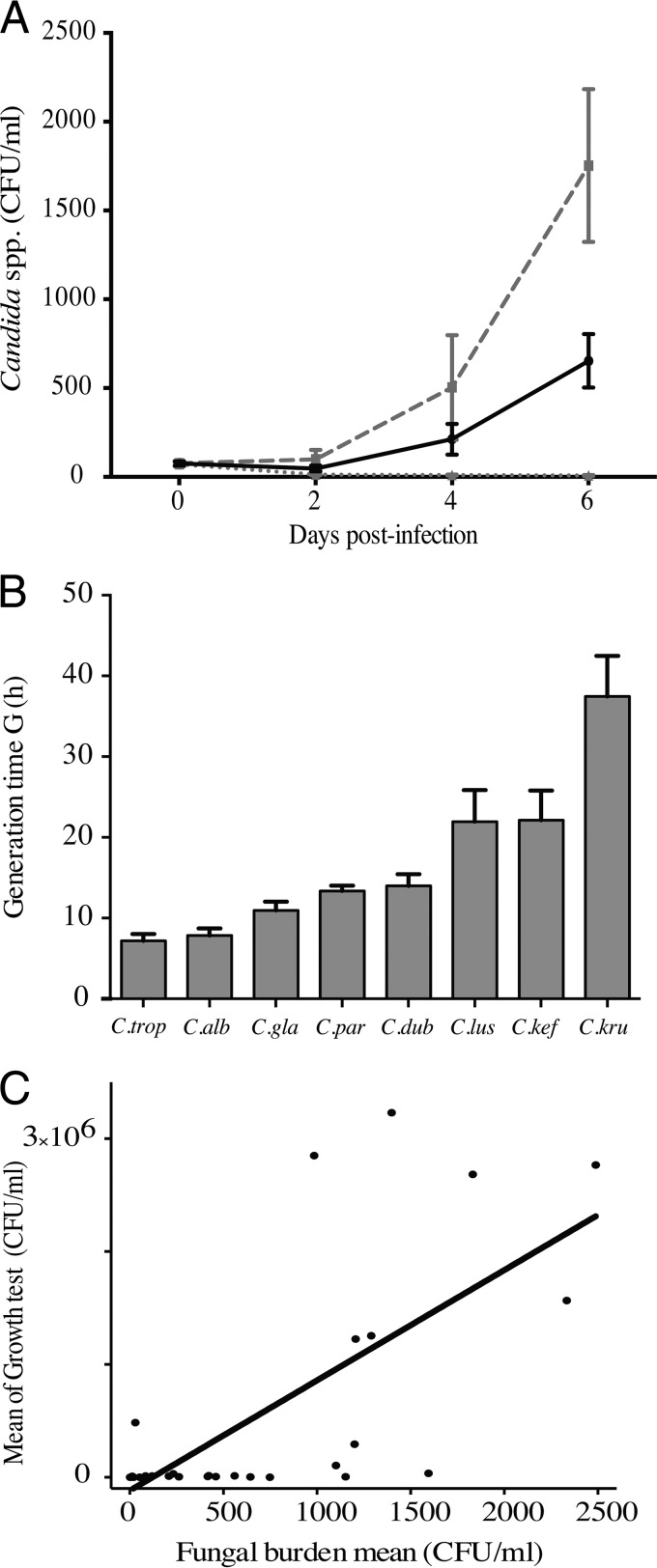
Dynamics of Candida proliferation within immune infiltrates. (A) The fungal burden was determined at 0, 2, 4, and 6 days postinfection. Data are the means ± SEM of the fungal burden of 32 clinical isolates of Candida from immune infiltrates of 10 subjects. The black line represents the mean ± SEM of the fungal burden of 32 clinical isolates of Candida and of 10 subjects. The gray dashed line represents the mean ± SEM of the fungal burden of C. albicans immune infiltrates, while the gray dotted line shows the mean ± SEM of the fungal burden of C. krusei immune infiltrates. (B) Generation time (G) and growth analysis of Candida species isolates. Shown are means + SEM for generation time for Candida species. (C) Significant positive correlation between mean of growth test after 6 days and mean of fungal burden on day 6 after infection.

After infection of human immune cells, the dynamics of the fungal burden was assessed by a colony counting method for up to 6 days. The results for the global fungal burden were expressed as the mean CFU per milliliter for the 32 clinical isolates and the 10 subjects. They showed a reduction of the fungal growth during the first 3 days postinfection for all Candida isolates. Then, the surviving yeasts were responsible for a significant and rapid increase in the fungal burden from the fourth to the sixth day postinfection (Fig. 3A). Interestingly, when it was analyzed at the species level, a strong variability in the fungal burden was identified on days 4 and 6 postinfection, reflecting a high interspecies variability of the phenomenon (Fig. 3A; Table 1).

**TABLE 1:** Dynamics of fungal burden within granuloma-like structures for Candida species

Species	Fungal burden mean, CFU/ml (SEM), on indicated day		
	2	4	6
C. albicans	98.7 (53)	503 (294)	1,753 (429)
C. dubliniensis	31 (19)	355 (300)	1,078 (403)
C. tropicalis	71 (35)	379 (177)	1,137 (351)
C. lusitaniae	29 (8.4)	118 (52)	604 (264)
C. parapsilosis	47 (19)	150 (85)	252 (118)
C. glabrata	71 (22)	171 (66)	380 (148)
C. kefyr	14 (5.4)	7.6 (3.2)	10 (5.7)
C. krusei	12.9 (3.9)	10 (2.8)	7.8 (2.3)

### Higher axenic Candida growth rates contributed to yeast persistence within immune infiltrates.

We next explored the influence of the growth rate of the Candida species on their ability to persist within immune infiltrates. The growth rate of 32 Candida isolates was measured under the same culture conditions as those for coculture studies. Figure 3A shows the generation time for each of the studied species. C. albicans and C. tropicalis showed short generation times (7.183 ± 0.834 h and 7.850 ± 0.881 h, respectively). In contrast, C. dubliniensis, C. glabrata, C. parapsilosis, and C. lusitaniae showed similar generation times (13.997 ± 1.447 h, 10.954 ± 1.078, 13.313 ± 0.677 h, and 21.933 ± 3.923 h, respectively). The generation times of C. kefyr and C. krusei were higher than for the other species (22.137 ± 3.675 h and 37.449 ± 5.037 h, respectively).

We further evaluated the relationship between growth rate and fungal burden in immune infiltrates. A correlation analysis was performed between the mean of the fungal burden during axenic growth tests and the mean of the fungal burden in immune infiltrates on day 6 of incubation for each of the 32 clinical isolates. Figure 3B shows a significant direct correlation between the growth rate of Candida species and their ability to persist within immune infiltrates (ρ = 0.775; *P* < 0.0001; *r*^2^ = 0.4974).

A two-way analysis of variance (ANOVA) highlighted remarkable differences in yeast proliferation profiles among these species; they were thus classified into three groups according to all of the parameters analyzed. Group A comprised Candida species showing high proliferation rates between days 4 and 6 postinfection. C. albicans, C. dubliniensis, and C. tropicalis actively escaped from immune infiltrates by forming blastoconidia, hyphae, or pseudohyphae. Human immune cells from the subjects poorly controlled this fungal proliferation (median fungal burden of 1,300 CFU/ml). The fungal burden dynamics of group B (C. lusitaniae, C. glabrata, and C. parapsilosis) was similar to those for group A. However, the mean of the fungal burden was significantly lower than for group A (400 CFU/ml). C. krusei and C. kefyr formed group C, characterized by the progressive decrease in the fungal burden from day 0 to up to 6 days postinfection. Immune infiltrates from those subjects controlled the fungal burden better over time (Fig. S1). We then analyzed whether clinical isolates had an effect on fungal burden dynamics for each Candida species. The results showed that the fungal burden dynamics within immune infiltrates was not significantly different between the four clinical isolates from each Candida species (Fig. S2 and S3).

### The histological analyses of Candida-induced immune infiltrates exhibit a cellular composition similar to granuloma-like structures.

**FIG 4:**
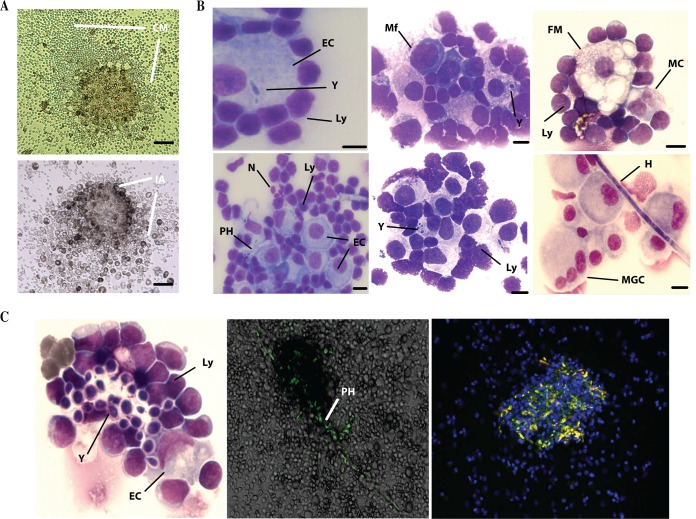
Histological examination of immune infiltrates induced by Candida spp. (A) Light microscopy observation of immune infiltrates and cell migration gradients (CM) 6 days postinfection. Cells were washed twice in PBS, and remaining compact inflammatory aggregates (IA) were analyzed. Scale bar: 50 μm. (B) May-Grünwald-Giemsa staining of immune aggregates 6 days after infection. Y, yeasts; PH, pseudohypha; H, hypha; Mf, activated macrophages; EC, large activated macrophages; MC, multinucleated cells with two nuclei; FM, foamy macrophages; MGC, multinucleated giant cells; N, neutrophils; Ly, lymphoid cells. Bars represent 20 μm. (C) Time-lapse and confocal microscopy examination of granuloma-like structures. Human immune cells were incubated with GFP-tagged C. albicans under the same coculture conditions as described in the text. Nuclear DNA was stained with Hoechst. Fluorescence-stained sections were examined under a Nikon A1 RSI microscope at a magnification of ×20 and constant Z-steps, and 3D images were processed with NIS elements version 3.21 and Volocity 3D image analysis software version 6.01.

Light microscopy observation of cocultures 6 days after challenge showed immune infiltrates with large zones of focal cell migration (CM). Isolation of these compact immune reactions after washing of nonassociated cells allowed the study of compact inflammatory aggregates (IA) (Fig. 4A). The composition of Candida-induced immune infiltrates was analyzed at a cellular level after May-Grünwald-Giemsa staining. Figure 4B shows high-magnification images of fungal and immune components. These structures were characterized by the organized presence of fungal elements surrounded by macrophages, some multinucleated cells having two nuclei, foamy macrophages, and rare multinucleated giant cells and neutrophils. Lymphoid cells were mainly present at the periphery, nearly in contact with macrophages. At this stage, Candida-induced immune infiltrates exhibited characteristics similar to those of granuloma-like structures. Time-lapse and confocal microscopy examination confirmed that inner fungal elements of C. albicans-GFP produced filamentation and disseminated from these granuloma-like structures (Fig. 4C).

### The abilities of human granuloma-like structures to control Candida infection were different between subjects.

**FIG 5:**
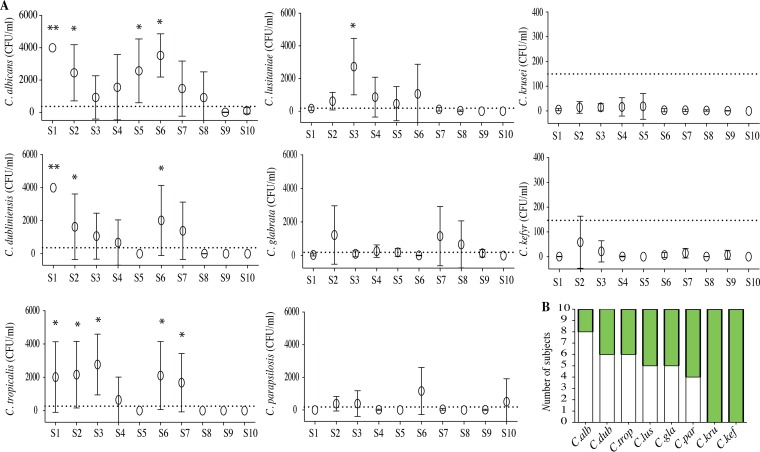
Interindividual variability of the response against Candida spp. The variability of the subjects' response against Candida infection was studied after infection of human peripheral blood mononuclear and polymorphonuclear cells with living yeasts from different Candida species. (A) Fungal burden 6 days postinfection. Results are expressed as the mean ± SEM of the fungal burden of 4 Candida clinical isolates from each species for each subject (S1 to S10). Dotted lines indicate the arbitrary cutoff of 100 CFU/ml. The mean ± SEM for each subject and each Candida species were compared to the mean ± SEM of species that controlled infection (mean of C. krusei and C. kefyr). *, *P* < 0.05; **, *P* < 0.001. (B) Number of subjects showing a persistent-infection (white bars) or controlled-infection (green bars) status.

We assessed whether the immune response of 10 healthy subjects had an impact on Candida proliferation within granuloma-like structures. Whereas immune cells from all subjects actively controlled all Candida proliferation during the first 3 days postinfection, an interindividual variability was observed in the response to the different Candida species between days 4 and 6 after challenge (Fig. 5). A cutoff was established to describe the ability of immune cells from subjects to control the infection or progressively be infected up to 6 days postinfection. The response of granuloma-like structures from subjects who exhibited a lower fungal burden than 100 CFU/ml on day 6 postinfection was referenced as controlled infection (CI). When the fungal burden was higher than 100 CFU/ml, granuloma-like structures from these subjects were defined as persistent infection (PI). The CFU per milliliter (mean ± standard error of the mean [SEM]) for each subject and each Candida species was compared to those for species that controlled infection (C. krusei and C. kefyr). As shown in Fig. 5A, structures poorly controlled the fungal burden of C. albicans, C. dubliniensis, C. tropicalis, C. lusitaniae, C. glabrata, and C. parapsilosis. For C. albicans, only two subjects were able to clear the infection. In contrast, granuloma-like structures from all of these subjects were able to control infection by C. krusei and C. kefyr, and six of them were able to clear the infection completely (Fig. 5B).

### The percentage and recruitment of phagocytic cells against Candida spp. were different between the controlled-infection and persistent-infection statuses.

**FIG 6:**
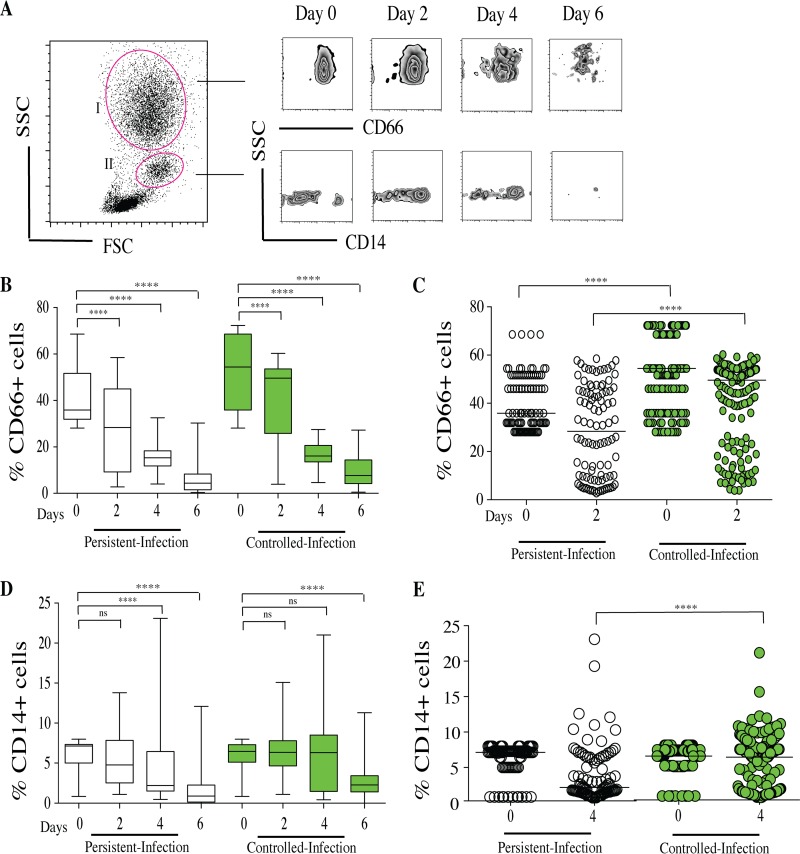
Characterization of CD66^+^ and CD14^+^ cell proportions within Candida granuloma-like structures over time. Peripheral blood mononuclear and polymorphonuclear cells from 10 healthy subjects were infected with 32 Candida clinical isolates for up to 6 days. The granuloma-like structures were collected from coculture plates at different time points and stained with a cocktail of fluorescence-conjugated antibodies specific to CD66 neutrophils and CD14 monocytes. (A) Representative flow cytometry analysis showing a side-scatter (SSC) versus forward-scatter (FSC) plot of granulocyte (I) and monocyte (II) selection. CD66^+^ cells were gated from region I, and CD14^+^ monocytes were gated from region II. (B) The proportions of CD66^+^ cells within granuloma-like structures are expressed as percentages of the total living cell compartment. Box plots depict median, minimum, and maximum percentages of CD66^+^ cells in persistent-infection and controlled-infection subjects. (C) Scatter plot of CD66^+^ proportions according to persistent-infection and controlled-infection status. (D) The proportions of CD14^+^ cells within granuloma-like structures are expressed as a percentage of the total living cell compartment. Box plots depict median, minimum, and maximum percentages of CD14^+^ cells according to persistent-infection and controlled-infection status. (E) Scatter plot of CD14^+^ cells proportions. *, *P* < 0.05; **, *P* < 0.001; ***, *P* < 0.0001; ****, *P* < 0.00001 (by one-way ANOVA with Tukey's multiple-comparison test) (*n* = 320). ns, not significant.

To assess whether the host immune cells had an impact on the dynamics of Candida fungal burdens, several flow cytometry assays of granulocyte subsets within granuloma-like structures over time were performed. CD66^+^ neutrophils from granulocyte region I and CD14^+^ monocytes were gated from monocyte region II. Results are expressed as the percentages of CD66^+^ and CD14^+^ from the total living cell compartment over time (Fig. 6A).

The kinetics of phagocytic cells after infection with each Candida isolate were similar between persistent-infection and controlled-infection granuloma-like structures. They were characterized by a progressive and significant reduction in the percentage of CD66^+^ cells on days 2, 4, and 6 compared to that on day 0 (Fig. 6B; Tables S1 and S2). Interestingly, in granuloma-like structures in which the infection was controlled, the initial mean percentage of CD66^+^ cells at the moment of the challenge (day 0) was significantly higher (55%) than in persistent-infection granuloma-like structures (40%) (Fig. 6C). Two days postinfection, the relative percentage of CD66^+^ cells reflected a 22% decrease when infection was controlled. However, during persistent infection, the CD66^+^ cell percentage was reduced more (35% decrease; *P* = 0.0002 versus controlled infection) (Fig. 6C).

In contrast to the case with CD66^+^ cells, there was no significant difference in the percentage of CD14^+^ on day 0 between persistent-infection and controlled-infection granuloma-like structures. The CD14^+^ cells also decreased over time after Candida infection. Interestingly, differences in the dynamics of CD14^+^ cells were observed between the persistent-infection and controlled-infection statuses (Fig. 6D). Four days after challenge, in persistent-infection granuloma-like structures, the percentage of CD14^+^ cells significantly decreased by 27% (*P* = 0.001 versus day 0). In contrast, this cell subset did not significantly decrease in controlled-infection granuloma-like structures (3%; *P* = 0.7 versus value for day 0). Overall, 4 days after infection, the relative proportion of CD14^+^ cells was significantly higher for the controlled-infection than for the persistent-infection status (Fig. 6E).

### CD56^+^ natural killer cells decreased in granuloma-like structures from subjects with controlled infection.

**FIG 7:**
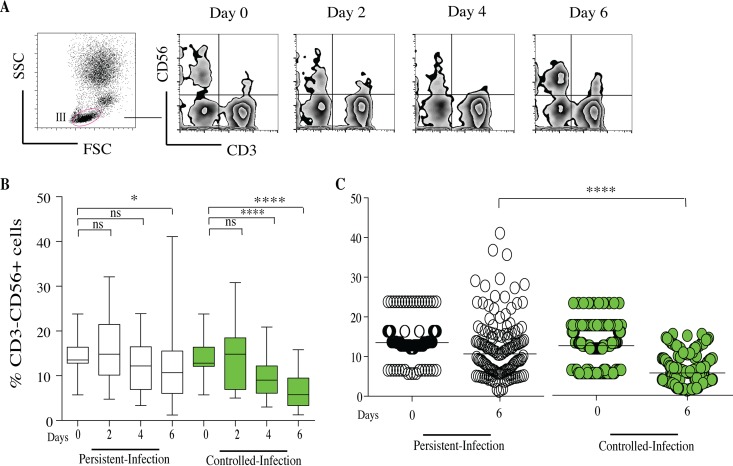
Characterization of CD56^+^ NK cells within Candida granuloma-like structures over time. Peripheral blood mononuclear and polymorphonuclear cells from 10 subjects were infected with 32 Candida clinical isolates for up to 6 days. The granuloma-like structures were collected from coculture plates at different time points and stained with a cocktail of fluorescence-conjugated antibodies specific to CD3 and CD56 lymphocytes. (A) Representative flow cytometry analysis showing SSC versus FSC plot of lymphocytes in section III. The cells were analyzed over time after gating on CD3 lymphocytes. Natural killer cells were gated as CD56^+^ from the CD3^−^ population. (B) The proportions of CD56^+^ cells within granuloma-like structures are expressed as percentage of the total CD3^−^ compartment. Box plots depict median, minimum, and maximum percentages of CD56^+^ cells in persistent-infection and controlled-infection granuloma-like structures. (C) Scatter plot of CD56^+^ NK cells proportions according to persistent- and controlled-infection status. *, *P* < 0.05; **, *P* < 0.001; ***, *P* < 0.0001; ****, *P* < 0.00001 (by one-way ANOVA with Tukey's multiple-comparison test) (*n* = 320).

CD56^+^ NK cells were gated from the human lymphocytes (region III). After gating on CD3 lymphocytes, CD56^+^ cells were gated from the CD3^−^ population (Fig. 7A). The proportions of CD56^+^ cells were expressed as the mean percentages of CD56^+^ cells from the total CD3^−^ compartment. The kinetics of CD56^+^ cells after infection with each Candida isolate were similar between persistent-infection and controlled-infection granuloma-like structures. There was no significant difference in the percentage of CD56^+^ cells on day 0 between the persistent-infection and controlled-infection statuses. The dynamics was characterized by a significant reduction in the percentage of CD56^+^ cells 6 days after challenge compared to day 0. Interestingly, in controlled-infection granuloma-like structures, the reduction in CD56^+^ cell percentage was significant from day 4 to day 6 (Fig. 7B). Six days postinfection, the relative proportions of CD56^+^ were lower in controlled-infection granuloma-like structures (mean of 6%) than in persistent-infection granuloma-like structures (11%; *P* = 0.00001).

### The high CD4^+^/CD8^+^ T lymphocyte ratio contributed to controlling infection within granuloma-like structures.

**FIG 8:**
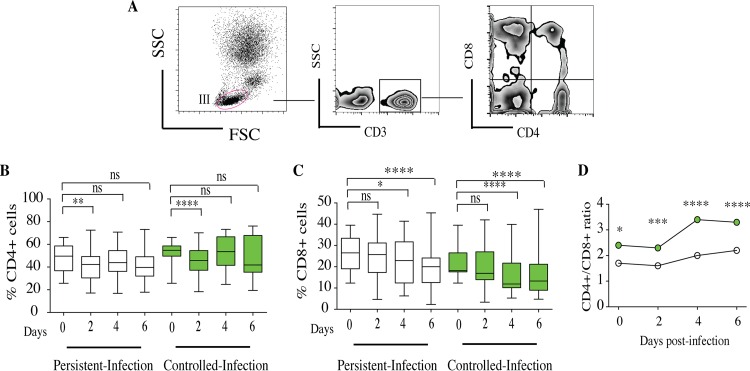
Characterization of CD4^+^ and CD8^+^ T cells within Candida granuloma-like structures over time. Peripheral blood mononuclear and polymorphonuclear cells from 10 subjects were infected with 32 Candida clinical isolates for up to 6 days. The granuloma-like structures were collected from coculture plates at different time points and stained with a cocktail of fluorescence-conjugated antibodies specific to CD3, CD4, and CD8 lymphocytes. (A) Representative flow cytometry analysis showing CD4^+^ and CD8^+^ cells over time after gating on CD3^+^ lymphocytes. (B and C) The proportions of CD4^+^ and CD8^+^ cells within granuloma-like structures are expressed as percentages of the total CD3^+^ compartment. Box plots depict median, minimum, and maximum percentages of CD4^+^ and CD8^+^ cells over time according to infection status. (D) Dynamics of CD4^+^/CD8^+^ cell ratio over time between persistent-infection and controlled-infection granuloma-like structures. *, *P* < 0.05; **, *P* < 0.001; ***, *P* < 0.0001; ****, *P* < 0.00001 (by one-way ANOVA with Tukey's multiple-comparison test) (*n* = 320).

T lymphocytes were gated from human lymphocyte region III. CD4^+^ and CD8^+^ T cells were gated from CD3^+^ T lymphocytes (Fig. 8A). The dynamics of CD4^+^ T cells were not significantly different over time between persistent-infection and controlled-infection granuloma-like structures (Fig. 8B). The infiltrating CD4^+^ cells were more abundant than CD8^+^ cells. The proportions of CD4^+^ T cells varied from 42% to 47% in persistent-infection granuloma-like structures. In controlled-infection granuloma-like structures, the CD4^+^ T cell proportions fluctuated between 45% and 53%. In contrast, the CD8^+^ T cell proportions significantly decreased between days 4 and 6 postinfection compared to those on day 0 in both granuloma-like structure types (Fig. 8C).

These kinetics of CD4^+^ and CD8^+^ T cells resulted in an increased CD4^+^/CD8^+^ ratio during the course of infection. Interestingly, this ratio remained significantly higher in controlled-infection than in persistent-infection granuloma-like structures (3.5 and 2.1, respectively) (Fig. 8D).

### Granuloma-like structures from persistent-infection status produced proinflammatory and anti-inflammatory cytokines.

Cytokines were quantified during granuloma-like structure formation by collecting supernatants over time. We investigated the release of gamma interferon (IFN-γ), interleukin-10 (IL-10), IL-4, IL-6, IL-17A, IL-17F, and tumor necrosis factor alpha (TNF-α). The cytokine profiles between persistent- and controlled-infection statuses were compared. In persistent-infection granuloma-like structures, the IFN-γ levels were low between days 2 and 4 compared to those of untreated controls on day 0, before reaching 5.8 pg/ml on day 6. In contrast, in controlled-infection granuloma-like structures, IFN-γ reached this level 2 days postinfection and then progressively decreased between days 4 and 6.

**FIG 9:**
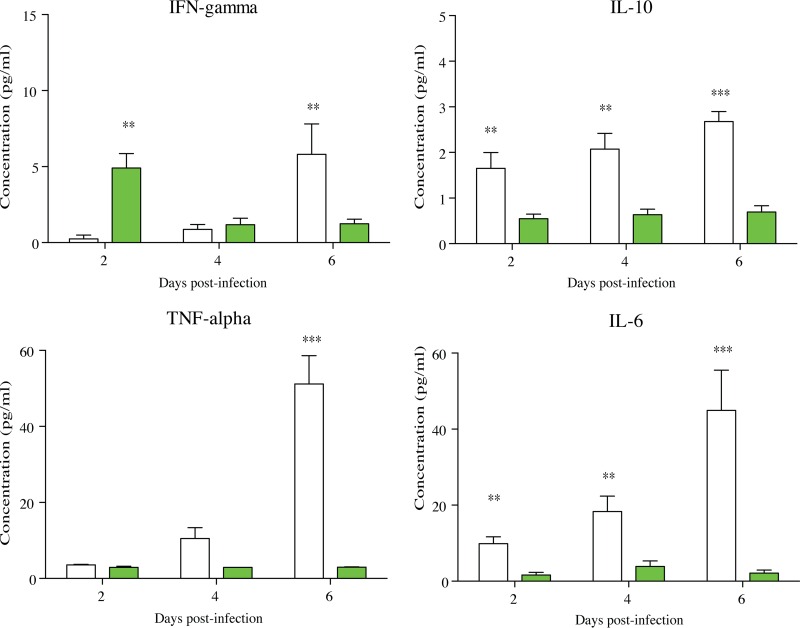
Cytokine profiles in persistent-infection and controlled-infection granuloma-like structures over time. Mean rates of IFN-γ, IL-10, IL-6, and TNF-α were compared between persistent-infection (white bars) and controlled-infection (green bars) granuloma-like structures by two-way ANOVA (α = 0.05). *, *P* < 0.05; **, *P* < 0.001; ***, *P* < 0.0001.

In persistent-infection granuloma-like structures, IL-6 levels progressively increased between days 2 and 6 postinfection (9.8 pg/ml on day 2 and 45 pg/ml on day 6). In the same way, TNF-α levels significantly increased over time (3.5 pg/ml on day 2 and 51 pg/ml on day 6). Interestingly, Il-10 secretion also progressively increased (Fig. 9, white bars).

In controlled-infection granuloma-like structures, the dynamics of these cytokines were not significantly different from those on day 0, and they were significantly lower than in persistent-infection granuloma-like structures (Fig. 9, green bars). IL-4, IL-17A, and IL-17F cytokines were not significantly detected over time.

### C. albicans, C. dubliniensis, and C. tropicalis induced a specific T lymphocyte response in persistent-infection granuloma-like structures.

**FIG 10:**
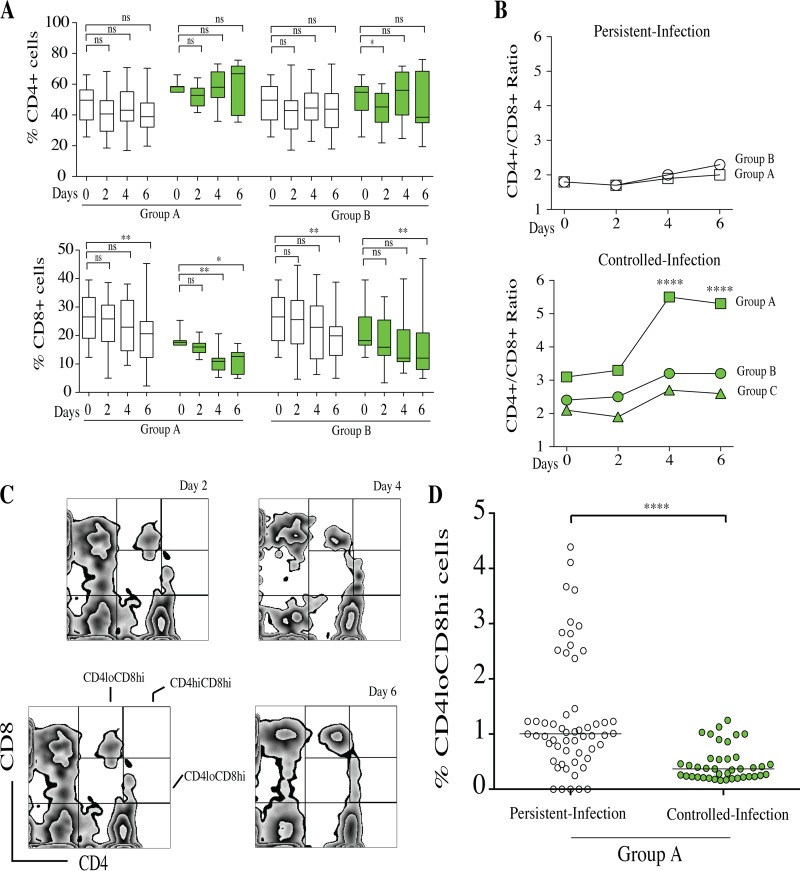
Characterization of CD4^+^ CD8^+^ doubly positive T cells within Candida granuloma-like structures from group A over time. (A) Proportions of CD4^+^ and CD8^+^ cells within granuloma-like structures are expressed as percentages of the total CD3^+^ compartment. Box plots depict median, minimum, and maximum percentages of CD4^+^ and CD8^+^ cells over time according to infection status (persistent [white boxes] and controlled [green boxes]) and Candida groups. (B) Dynamics of CD4^+^/CD8^+^ ratio over time between persistent-infection and controlled-infection granuloma-like structures. (C) Representative flow cytometry analysis showing CD4^+^ and CD8^+^ cells after gating on CD3^+^ lymphocytes. CD4^lo^ CD8^hi^, CD4^hi^ CD8^hi^, and CD4^hi^ CD8^lo^ cells were analyzed in the upper right quadrant. (D) The proportions of CD4^lo^ CD8^hi^ cells within granuloma-like structures are expressed as percentage of the total CD4^+^ CD8^+^ doubly positive T cell compartment. *, *P* < 0.05; **, *P* < 0.001; ***, *P* < 0.0001; ****, *P* < 0.00001 (by one-way ANOVA with Tukey's multiple-comparison test) (*n* = 120).

The link between Candida species and the establishment of a specific immune response within granuloma-like structures was examined. The composition of immune granuloma-like structures was analyzed according to groups A, B, and C. The dynamics of CD66^+^, CD14^+^, and CD56^+^ cells were not significantly different between Candida groups (Tables S1, S2, and S3), suggesting that these immune profiles were independent of the infecting Candida species. However, the analysis of CD4^+^ and CD8^+^ T lymphocyte dynamics showed specific signatures against C. albicans, C. dubliniensis, and C. tropicalis challenges (group A) compared to groups B and C (Fig. 10).

In persistent-infection granuloma-like structures from groups A, B, and C, the dynamics of CD4^+^ T cells was essentially unchanged during the course of infection, while CD8^+^ T cells significantly decreased. Although there were more CD4^+^ T cells in persistent-infection granuloma-like structures from groups A and B, the T cell ratio was close to 2. In controlled-infection granuloma-like structures, the CD4^+^ T cells were somewhat more abundant during the 4th and 6th days postinfection, while CD8^+^ T cells were significantly reduced over time (Fig. 10A). The relative reduction in CD8^+^ T cells induced a temporary increase in the CD4^+^/CD8^+^ ratio. Interestingly, in controlled-infection granuloma-like structures from group A, the CD4^+^/CD8^+^ ratio was significantly higher than groups B and C (Fig. 10B).

To investigate the origin of the relative reduction in CD8^+^ T cell proportions 6 days postinfection, the CD4^+^ CD8^+^ doubly positive T cells in the CD3^+^ compartment were analyzed. Figure 10C depicts a representative analysis of double-positive T cells in the upper right quadrant. The proportions of CD4^lo^ CD8^hi^, CD4^hi^ CD8^hi^, and CD4^hi^ CD8^lo^ cells were determined over time after Candida infection. The average proportions of CD4^hi^ CD8^hi^ and CD4^hi^ CD8^lo^ cells were not significantly different over time between persistent-infection and controlled-infection statuses (Tables S1, S2, and S3). Interestingly, a significantly higher proportion of CD4^lo^ CD8^hi^ T cells infiltrating granuloma-like structures was found for the persistent-infection status than for the controlled-infection status (Fig. 10D).

### PMN depletion from granuloma-like structures is crucial for the persistent-infection status.

**FIG 11:**
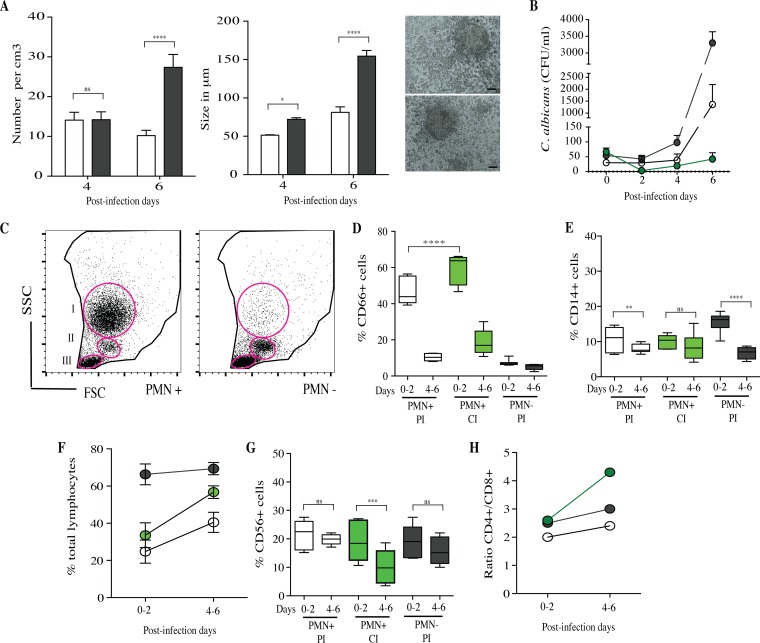
Characterization of PMN-depleted granuloma-like structures. (A) Number and size of granuloma-like structures on days 4 and 6 after infection by C. albicans species. Black bars, PMN-depleted conditions; white bars, nondepleted control conditions. Right images depict representative immune infiltrates observed under light microscopy 6 days after challenge under PMN-depleted conditions. (B) Dynamics of C. albicans CFU per milliliter over time. Shown are means ± SEM of the fungal burdens of C. albicans clinical isolates in PMN-depleted granuloma-like structures showing a PI status (gray circles), in PMN^+^ granuloma-like structures showing a PI status (white circles), and in PMN^+^ granuloma-like structures showing a CI status (green circles). (C) Representative flow cytometry analysis showing SSC-versus-FSC plot of granulocyte (I), monocyte (II), and lymphocyte (III) selection under PMN-depleted (PMN^−^) and nondepleted control (PMN^+^) conditions. CD66^+^ cells were gated from region I; CD14^+^ monocytes were gated from region II. CD3^−^ CD56^+^, CD3^+^ CD4^+^, and CD3^+^ CD8^+^ cells were gated from region III. (D and E) The proportions of CD66^+^ and CD14^+^ cells within granuloma-like structures are expressed as percentages of the total living cell compartment. Box plots depict median, minimum, and maximum percentages of cells in PMN^+^ PI, PMN^+^ CI, and PMN^−^ PI. (F) Total lymphocyte proportions according to PMN^+^ PI (white circles), PMN^+^ CI (green circles), and PMN^−^ PI (gray circles) statuses. (G) Proportions of CD56^+^ cells within granuloma-like structures over time in PMN^+^ PI, PMN^+^ CI, and PMN^−^ PI outcomes. (H) Dynamics of CD4^+^/CD8^+^ ratio over time. *, *P* < 0.05; **, *P* < 0.001; ***, *P* < 0.0001; ****, *P* < 0.00001 (by one-way ANOVA with Tukey's multiple-comparison test) (*n* = 40).

To investigate the role played by polymorphonuclear leukocytes (PMN) during the first 2 days postinfection and the relationships with the dynamics of cellular interactions, the granuloma-like structure assay was performed in their absence. PBMC were infected with C. albicans clinical isolates, and granuloma-like structure formation over the time was observed in all of subjects. The number and size of these structures were significantly higher when PMN were depleted on day 0 than those under PMN^+^ control conditions (Fig. 11A). The mean CFU per milliliter within granuloma-like structures was higher under PMN^−^ conditions than under PMN^+^ control conditions. However, the dynamics of CFU per milliliter overlapped substantially between the two groups. As previously shown, two subjects were able to clear infection by all of the C. albicans clinical isolates (Fig. 11B).

For flow cytometry analyses, CD66^+^ and CD14^+^ cells and total lymphocytes were gated from the living cell compartment (regions I, II, and III, respectively). Results were indicated in percentages of cells from the total living cells (Fig. 11C). The mean percentages of CD66^+^, CD14^+^, and lymphocytes on day 0 of infections were, respectively, 7%, 16%, and 66%. Due to the significant depletion of CD66^+^ PMN cells on day 0 of infection, the proportions of CD14^+^ and total lymphocytes were significantly higher than under PMN^+^ control conditions. As previously shown under PMN^+^ conditions, the proportion of CD66^+^ cells was significantly higher after 2 days in granuloma-like structures in which infection was controlled (PMN^+^, CI) than under persistent-infection status (PI). Granuloma-like structures under PMN^−^ conditions showed mean percentages of CD66^+^ cells less than 7% at 6 days postinfection (Fig. 11D).

Even if a higher proportion of CD14^+^ cells was present during the first 2 days of infection under PMN^−^ conditions, the mean percentage of these cells was significantly decreased, by 56%, between days 4 and 6. These results fit with the dynamics of CD14^+^ cells in persistent-infection granuloma-like structures (Fig. 11E). The infiltrating total lymphocytes were significantly more abundant under PMN^−^ conditions; the percentages varied between 66% and 71% over the duration (Fig. 11F). Similar to the case with persistent-infection outcomes, the dynamics of CD56^+^ cells under PMN^−^ conditions was characterized by a lower reduction of this cell subset on days 4 and 6 than with controlled-infection outcomes (Fig. 11G). Despite the relative abundance of total lymphocytes, the ratio of CD4^+^ to CD8^+^ cells between days 4 and 6 was significantly lower than for the controlled-infection status (Fig. 11H).

## DISCUSSION

The understanding of the complex relationships between human and Candida has known tremendous progress in the last decades (14, 41, 42). The efficiency of human innate and adaptive immunity in controlling Candida infections is underscored by the wide range of Candida species, by their pathogenic adaptations (morphological switch), by the diversity of clinical manifestations and by the variety of pathophysiological niches (43). The concept of a damage response framework (DRF) from the perspective of candidiasis clearly defined how much human infections are a result of Candida-mediated damage, human-mediated damage, or both (13). From this context, it is clear that fungal factors interact closely with multiple human immune cells at a local tissue level (31). However, when local immune responses are impaired, this usually leads to dissemination. In addition to C. albicans, which is the most common cause of bloodstream infections, other Candida species have become prevalent. While the short-time interaction of C. albicans with human immune cells has been well investigated, studies of other species need to be improved (44).

The goal of the present study was to examine, at a cellular scale, Candida spp. and human immune cells in a long-term interaction. After infection of human immune cells with clinically relevant Candida species, we found variations in the cellular composition and cytokine environment of immune infiltrates during the course of infection. To our knowledge, this is the first reported experiment showing that clinically relevant Candida species induced such immune infiltrates after a long-term interaction with human cells. These local immune responses could represent the main interface between the fungi and the host.

Thirty-two clinical isolates from eight clinically relevant Candida species were used to infect human peripheral mononuclear and polymorphonuclear immune cells from immunocompetent subjects. These species, primarily found in infected tissues, undergo a combination of yeast, hypha, and pseudohypha morphological switches. In this study, we have demonstrated that interaction of Candida spp. and human immune cells from subjects without any apparent immunodeficiency is dependent on the fungal species and the immune cells with which they interact. Morphometric and histological analyses highlighted for the first time that immune infiltrates are induced by Candida spp. and that they shared a cellular composition similar to granuloma-like structures. Due to the broad variability of granuloma reactions induced against infectious diseases, their classification remains complex. Some granuloma structures are composed by activated macrophages surrounding a necrotic region with a ring of T and B lymphocytes. Other types of granulomas include nonnecrotizing granulomas (which activated epithelioid macrophages with some lymphocytes), necrotic neutrophilic granulomas, and completely fibrotic granulomas. Our data are in line with the histological description of nonnecrotizing granulomas formed during bacterial infections, such as during M. tuberculosis infection (37, 39, 45). Moreover, the technical manipulation and isolation of these complex structures were similar to those implemented for mycobacterial *in vitro* granulomas. Similar models have been useful to investigate and recapitulate other mycobacterial, protozoan, helminthic, and fungal pathophysiologies, for which granuloma formation is a hallmark of pathology (38, 46–52). In a previous work, we have validated an *in vitro* multicellular interaction model with C. albicans (40).

Considering that the granulomatous inflammatory reaction during invasive candidiasis is rarely described (24–27), few studies have addressed the question of long-term local immune responses. Our experimental data suggest that multicellular immune infiltrates are formed after Candida infection, sharing characteristics of a granuloma-like reaction. These reactions deserve more investigation, since they are important interfaces between the host and Candida. Moreover, the accurate relationship between granuloma-like dynamics and chronic candidiasis pathophysiology needs further studies. Microabscesses that are usually observed during disseminated Candida infections are scattered foci of necrosis surrounded by polymorphonuclear leukocytes, macrophages, and epithelioid cells. In light of our observations, it is not clear, however, how these structures could be the consequence of late immune infiltrates. Furthermore, the translational relevance of this granulomatous reaction is in agreement with murine models of hematogenously disseminated candidiasis in which infection occurs in hosts with weak or normal immune responses (29, 30).

We observed that fungal growth was controlled during the first 2 days after infection in the same way for all the Candida clinical isolates. However, substantial differences were found between species in terms of their resulting fungal proliferation 6 days after challenge. Three different groups of Candida species were identified, by taking into account fungal proliferation and host cellular response characteristics (number and size of induced granuloma-like structures, the resulting fungal burden, and the growth rates 6 days after challenge). Group A (C. albicans, C. dubliniensis and C. tropicalis) was characterized by high proliferation rates from day 4 to day 6. C. lusitaniae, C. parapsilosis, and C. glabrata (group B) also formed granuloma-like structures, but their proliferation rates were lower than for group A. Group C comprised C. krusei and C. kefyr, which showed longer generation times than the other species and were progressively cleared from granuloma-like structures over time. These observations are consistent with phylogenetic studies showing that C. albicans, C. dubliniensis, and C. tropicalis are closely related and form hyphae and pseudohyphae (53). Moreover, the ability of Candida species to form hyphae constituted a virulence factor. In addition, C. lusitaniae and C. parapsilosis, which are found in infected tissues as a combination of pseudohyphae and yeasts, have a lower virulence and rarely or never form hyphae. Based on the dynamics of the fungal burden, two infection outcomes were identified: a controlled-infection (CI) status comprised the granuloma-like structures from subjects with a fungal burden lower than 100 CFU/ml on day 6 postinfection, while the persistent-infection (PI) status comprised granuloma-like structures from subjects whose fungal burden was higher than 100 CFU/ml.

In the second part of this study, the dynamics of cellular interactions between controlled-infection and persistent-infection statuses were compared. As the role of phagocytic cells in Candida infection has been clearly demonstrated, the analyses began by taking into account the dynamics of these cells in these human-like environments. Candida species are actively recognized by monocytes/macrophages (5). Neutrophils are also essential for the early clearance of Candida yeasts and hyphae (54). These cell types are crucial in the innate response against systemic C. albicans infections, underscored by the fact that prolonged neutropenia and macrophage disruption are risk factors of invasive candidiasis (55). Our results confirmed the essential role of phagocyte cells during the first steps and the outcome of infection. Hence, significantly higher percentages of CD66^+^ neutrophils were found on day 0 when infection was controlled. Then, in both controlled and persistent infections, CD66^+^ cell percentages drastically decreased between day 0 and day 6 after challenge. These results could be due to the natural death of neutrophils under our experimental conditions or to their destruction after killing of Candida yeasts and hyphae by breaking down and releasing their nuclear content as neutrophil extracellular traps (NETs) (56–58). Therefore, 2 days after challenge, CD66^+^ neutrophils still remained high within granuloma-like structures for the controlled-infection status. Studies involving neutrophils are challenging considering their half-life in the circulation of approximately 6 h. However, recent studies have demonstrated that during inflammation, neutrophils become activated and their longevity is increased in order to prime immune responses (59, 60).

The CD14^+^ proportions did not differ between controlled and persistent infections at the time of challenge. However, in granuloma-like structures in which infection was controlled, CD14^+^ macrophages remained abundant over 4 days after infection, whereas they were reduced when infection persisted. Previous work with Histoplasma capsulatum granuloma-like structures also provided evidence that the proportion of macrophages decreases at later time points (52). Studies addressing the role of phagocytes in fungal infections have shown that the killing of macrophages by Candida spp. is an evading mechanism by which the fungus can replicate in phagocytes and then disseminate (61). Overall, it is clear from our results that higher proportions of CD66^+^ neutrophils at the beginning of infection and a better resistance of macrophages to killing by Candida over 4 days after infection are significantly related to controlled-infection outcomes. This argument could be supported by the fact that monocytes can increase neutrophil phagocytic recruitment and may play a crucial role in controlling disseminated fungal infection (62).

In accord with the classic cytotoxic functions of NK cells, which respond to invading pathogens by recognizing infected phagocytes and inducing direct cell death (63), our findings seem to indicate that such NK cell properties act at the early stages of infection and are progressively cleared in controlled-infection granuloma-like structures. A recent study showed that C. albicans established contact with human NK cells before engaging in interactions resembling phagocytosis (17). Moreover, these results are supported by other studies showing that the depletion of NK cells in immunocompetent mice does not adversely affect survival after infection with C. albicans (18). Additionally, human NK cells exert anti-Candida direct effector functions or act indirectly by priming neutrophil candidacidal activity (17).

Neutrophils present antigens for T cell activation, leading to the recruitment of CD4^+^ T cells that support the generation of an optimal CD8^+^ T cell population (64). Previous studies have shown that CD4^+^ T cells in mice are significantly produced after stimulation with Candida antigens (65). It has been proposed that specific protection against a variety of mycoses corresponds to the activation of CD4^+^ T cells (66). Other studies have suggested a role for CD8^+^ T cells in the elimination of C. albicans from liver and gastrointestinal mucosa (67). Growth of C. albicans hyphae could also be inhibited by activated CD8^+^ T cells (68). The relative contribution of CD4^+^ T cells to the recruitment of CD8^+^ T cells is supported by clinical studies showing a particularly high incidence of oropharyngeal candidiasis in HIV patients with low CD4^+^ T cells (69, 70). Our results suggested that CD4^+^ and CD8^+^ T lymphocytes were significantly recruited into granuloma-like structures. Whereas the CD4^+^ T cell percentage fluctuated over time, the CD8^+^ T cell percentage progressively decreased. Similarly to the case with other studies of disseminated candidiasis, the percentages of CD4^+^ T cells were higher than those of CD8^+^ T cells at all time points tested (71, 72).

Interestingly, the results obtained in this study also highlighted that the CD4^+^/CD8^+^ ratio was higher in controlled-infection than in persistent-infection granuloma-like structures. This difference was attributable to a decrease in CD8^+^ T cell proportions. From these analyses, both T cells and CD56^+^ NK cells could contribute to the IFN-γ milieu that we observed during the earlier stages of granuloma-like structure formation in the controlled-infection status.

To our knowledge, this study provides, for the first time, evidence that CD4^+^ CD8^+^ doubly positive (DP) T cells infiltrate human granuloma-like structures of Candida spp. during the late stages after challenge. However, the functions and phenotypic characteristics of this specific T cell subset during candidiasis are still unknown. Total CD4^+^ CD8^+^ lymphocytes have previously been observed during vaginal inoculation of C. albicans into estrogen-conditioned mice (73). Another study observed that mouse immunization with mannan–human serum albumin conjugates from C. dubliniensis induced a significant increase of CD4^+^ CD8^+^ T cells (74). Previous studies reported that CD4^lo^ CD8^hi^ T cells increase in patients with chronic viral infections, autoimmune diseases, and cancer (75). Taken together, our results showed specific signatures in the fluctuation of CD4/CD8 cell ratios between Candida species. Proportions of CD4^lo^ CD8^hi^ T cells were significantly higher in granuloma-like structures for the persistent-infection status than in those for the controlled-infection status when cells were infected by C. albicans, C. dubliniensis, and C. tropicalis.

Taken together, our data indicated that Candida granuloma-like structures lead to a controlled- or persistent-infection status. High proportions of CD66^+^ neutrophils at the beginning of challenge characterized a controlled-infection status. Moreover, CD56^+^ NK cells and the elevated CD4^+^/CD8^+^ T cell ratio were correlated with early production of IFN-γ. Additionally, local IFN-γ could activate CD14^+^ macrophages to produce reactive radicals essential for Candida eradication. The early production of cytokines such as IFN-γ was also observed in correlation with C. albicans clearance from the oral mucosa of BALB/c mice (76). Moreover, variations in cytokine production resulted in a persistent phenotype. In the persistent-infection status, cytokine production was delayed and was composed of a high ratio of proinflammatory versus anti-inflammatory cytokines, such as IL-6, IFN-γ, TNF-α, and IL-10. Under our conditions, the production of IFN-γ was suboptimal and delayed, suggesting an inability of the host to eliminate the infection. This correlates with the delayed and lower production of cytokines in mouse models susceptible to candidiasis (76). As we compare our study to other *in vitro* studies, the detection of low cytokine concentrations under our culture conditions could be explained by methodological differences such as the antigenic stimulation, immune cell concentrations, and cytokine turnover (77). Nevertheless, PMN depletion studies indicated that PMN not only are major effectors of during acute Candida infection but also could be a determinant during chronic inflammatory conditions and adaptive responses against this fungal pathogen. By defining the precise cellular orchestration during Candida infections, it would be possible to manipulate these responses in the infection site to enhance their effector functions. These granuloma-like structures seem to be relevant for the pathophysiology of candidiasis and could enable the exploration of novel antifungal strategies.

## MATERIALS AND METHODS

### Preparation of Candida blastoconidium suspensions.

Eight Candida species (C. albicans, C. dubliniensis, C. tropicalis, C. lusitaniae, C. glabrata, C. parapsilosis, C. krusei, and C. kefyr) were tested for the ability to induce immune infiltrates. Four clinical isolates from each species were analyzed. The 32 Candida clinical isolates were obtained from the MycoBank of the Parasitology and Medical Mycology Department, Nantes, France. Clinical isolates were sown on potato dextrose agar (PDA) and incubated overnight at 30°C. For *in vitro* granuloma-like structure experiments, yeast cells were counted and suspended at different concentrations in RPMI 1640 (Sigma) supplemented with 8% human serum (HS).

### Candida and human leukocyte cocultures.

Blood samples were obtained from 10 healthy subjects by venipuncture at the Etablissement Français du Sang, Pays de la Loire, France. Blood was processed within 2 h of collection. Peripheral blood mononuclear cells (PBMC) and polymorphonuclear leukocytes (PMN) were subsequently separated by dextran sedimentation (dextran 500, 8% [wt/vol]; density, 1.113 ± 0.001 g/ml), followed by gradient centrifugation (dextran/blood ratio of 1:1). Plasma was removed and the first higher band of PBMC was separated and suspended in RPMI 1640–8% HS. The remaining lower band of PMN (>98%) was removed and suspended in the same medium. Cell suspensions were washed twice in RPMI 1640–8% HS by centrifugation. Fresh cell fractions were enumerated by cell counting and pooled following their basal proportions. The ratio of PMN over monocytes varied between 10:1 and 8:1 depending on each subject count. The range of monocyte percentages of 10 donors varied between 4% and 12%. The range of PMN percentages was between 45% and 65%. Total leukocytes (pooled PMN and PBMC) were adjusted to a final concentration of 2.5 × 10^6^ cells/ml and cultured in RPMI 1640–8% HS in 24-well tissue culture plates (around 2.0 × 10^5^ cells were monocytes). Freshly isolated cells were immediately infected with 32 Candida clinical isolates from eight species at a monocyte/blastoconidium ratio of 2,000:1. Cells were incubated à 37°C and 5% CO_2_. Uninfected leukocytes were used as a negative control of cellular aggregation. The fungal burden within compact immune infiltrates was determined by CFU calculation, and human immune cell composition over time was analyzed by flow cytometry on days 0, 2, 4, and 6 postinfection. The total number and viability of cells at each time point were assessed by cell counting in the presence of 0.5% eosin. Immune infiltrates were counted under light microscopy (×50) at 4 and 6 days postinfection. Their sizes in micrometers were also determined.

### PMN depletion from Candida and immune cell cocultures.

We assessed the effect of PMN exclusion at day 0 of infection on the dynamics of immune infiltrate formation and cell composition over the time. Differential counts on the mononuclear cell population showed between 1 to 7% granulocytes after separation. After washing twice in RPMI 1640–8% HS, PBMC were adjusted to a final concentration of 2.5 × 10^6^ cells/ml and cultured in RPMI 1640–8% HS. Fresh preparations were infected with C. albicans clinical isolates from at a monocyte/blastoconidium ratio of 2,000:1. PMN-depleted immune infiltrates were analyzed as described above and compared to those under PMN^+^ conditions. The ratio of PMN to monocytes for these control conditions was 8:1.

### CFU assay.

The Candida growth (CFU) was measured by counting the living yeasts with a colony-counting technique at different time points (0, 2, 4, and 6 days postinfection). After elimination of the supernatant, cells were washed twice in RPMI 1640–8% HS and cell layers were gently scraped. Cell suspensions were homogenized by pipetting, and several dilutions of each well (two wells per point) were plated on PDA plates. After incubation for 24 to 48 h à 30°C, Candida colonies were counted. Data were expressed in CFU per milliliter and corresponded to the fungal burden of both yeasts within the granuloma-like structures and, for filamentous species, hypha formation.

### Growth test.

Growth rates of 32 Candida isolates were tested under the same culture conditions as used for the study of granuloma-like structures (RPMI 1640 plus 8% HS, 37°C, and 5% CO_2_). One culture plate was used for each clinical isolate, and fungal multiplication was followed by assessing living yeasts in CFU at 24 h, 48 h, 3 days, 4 days, and 6 days. The maximum growth rate (μ_max_) and the generation time were calculated.

### Histological analyses.

Immune infiltrates were collected on days 4 and 6 after infection. After elimination of the supernatant, cell layers were washed twice in phosphate-buffered saline (PBS) supplemented with 2% fetal bovine serum (FBS). Remaining compact immune-associated cells were gently scraped without dispersion in PBS–2% FBS and plated on glass slides by centrifugation at 500 rpm in a Cytospin centrifuge (Cytobuckets S; Jouan). Slides were stained with May-Grünwald-Giemsa staining, and fungal and immune cells associated with immune infiltrates were analyzed by light microscopic identification.

### Video imaging.

For coculture analyses by video imaging, PBMC were infected with CAI4 C. albicans cells containing the pACT1-GFP fusion protein. Ten-milligram quantities of pACT1-GFP and pGFP fluorescence negative-control plasmids were linearized by digestion with BglII and used to transform C. albicans by electroporation. Single-copy integrants at the RPS10 locus were selected as previously described by Barelle et al. (78). Single colony transformants from minimal SD (single dextrose) medium containing 2% glucose were inoculated into 1 ml of synthetic complete medium containing Casamino Acids in order to induce ACT1-GFP expression. The cells were incubated for 2 h at 30°C to reach maximum fluorescence, collected by centrifugation at 3,000 × g for 10 min, analyzed to find the expression level under fluorescence microscopy (Leica Microsystems, Nanterre, France), and then used to infect human PBMC at a multiplicity of infection (MOI) of 200:1. This MOI was chosen in order to increase the probability of finding and recording the formation of immune infiltrates. The cells were illuminated at day 0 and every 10 min over 72 h of incubation with a 300-W xenon lamp equipped with a 488-nm excitation filter. Emission at 515 nm was used to analyze C. albicans fluorescence with a Leica DMI6000B CoolSnap HQ2 camera (Roper, Tucson, AZ) and processed with Metamorph imaging software version 7.7.4.0 (Universal Imaging, Downington, PA).

### Confocal microscopy.

Human immune cells were incubated with GFP-tagged C. albicans under the same coculture conditions as described above. Cells were incubated in Lab-Tek slides for 6 days at 37°C and 5% CO_2_. After being washed twice in PBS and fixed with 4% paraformaldehyde for 30 min, cells were permeabilized with 100% acetone. Nonspecific binding sites were blocked with 1% bovine serum albumin (BSA) in PBS for 30 min. Rhodamine-conjugated phalloidin (Wako; Osaka, Japan) was added at a 1:600 dilution to stain actin filaments for 30 min at room temperature. Nuclear DNA was stained with Hoechst in PBS for 1 min. Slides were air dried and mounted with Vectashield medium. Fluorescence-stained sections were examined under a Nikon A1 RSI microscope with a magnification of ×20 at constant Z-steps of 1 μm. The laser confocal system comprised a 65-mW multi-Ar laser. Three-dimensional (3D) images were processed with NIS elements version 3.21 (Nikon Instruments Inc.) and Volocity 3D image analysis software version 6.01 (PerkinElmer).

### Flow cytometry.

After elimination of the cell culture supernatant, granuloma-like structures were washed twice in PBS at 37°C in order to eliminate nonadherent cells and keep only granuloma-like structures. The total number and viability of cells at each time point were assessed by cell counting in the presence of 0.5% eosin. Granuloma-like structures from two well plates per condition were gently scraped, pooled, and dispersed by pipetting. After single living cell gating, the mean percentages of viable cells varied between 75 and 95% for all donor samples. The same was done with 2 control wells for each day tested (2, 4, and 6 days). The cells were suspended in 250 μl of PBS–1% BSA and stained with a cocktail of fluorescence-conjugated antibodies in PBS–1% BSA. The antibodies were specific to CD3-VioBlue (clone BW264/56; MACS Miltenyi Biotec), CD4-fluorescein isothiocyanate (FITC) (clone VIT4; MACS Miltenyi Biotec), CD8-phycoerythrin (PE) (clone BW135/80; MACS Miltenyi Biotec), CD56-allophycocyanin (APC) (clone AF12-7H3; MACS Miltenyi Biotec), CD14-APC-Vio770 (clone TÜk4; MACS Miltenyi Biotec), and CD66abce-PE-Vio770 (clone TET2; MACS Miltenyi Biotec). Stain specificity was verified with isotype-matched control antibodies FITC-conjugated mouse IgG1, VioBlue-conjugated mouse IgG1, APC-conjugated mouse IgG1, and PE-conjugated mouse IgG2. Cells were incubated for 1 h at 4°C in the dark, washed twice with PBS, and analyzed by flow cytometry. After gating on CD3 lymphocytes, the CD3^+^ population was separated into CD4^+^ and CD8^+^ T cells. Natural killer cells were gated out from the CD3^−^ population. CD66^+^ neutrophils and CD14^+^ monocytes came from granulocyte and monocyte regions. All data were acquired using a FACSCanto II instrument (BD Biosciences) and analyzed with FlowJO software version 9.4.10 (Tree Star Inc.) and DIVA software version 6.2 (BD Biosciences) to separate the different cell subsets constituting granuloma-like structures.

### Cytokine assay.

One milliliter of supernatant was removed for each clinical isolate and for the control at 2, 4, and 6 days postinfection. Samples were conserved at −80°C. Different cytokines (IFN-γ, TNF-α, IL-2, IL-4, IL-6, IL-10, IL-17A, IL-17F, and IL-22) were quantified with a ProcartaPlex multiplex immunoassay kit (Affymetrix; eBioscience) on a MAGPIX system (Luminex) according to the manufacturer's instructions. One multiplex suspension bead array immunoassay enabled the simultaneous measurement of cytokine supernatant levels (according to the standard protocol). Standard curves of each analyte were generated using the reference analyte concentration supplied by the manufacturer. Each sample was measured twice. Cytokine concentrations were calculated using ProcartaPlex Analyst 1.0 software (Affymetrix; eBioscience).

### Statistical analyses.

Statistical analyses were all carried out with Prism V6.0a software (GraphPad Software). The fungal burdens 6 days after challenge with each Candida species were compared using a two-way ANOVA test with Tukey's correction for multiple comparisons. The fold increase of fungal burdens and the growth rates were calculated in percentages and added to the multiple-comparison analyses. The size and number of induced granuloma-like structures were also compared between species with a one-way ANOVA with Tukey's correction for multiple comparisons. The D'Agostino & Pearson Omnibus normality test was performed on all data. Whether data were normally distributed or not, parametric *t* tests and two-way ANOVA were used when comparing two or more groups. For correlation, nonparametric Spearman's ρ was calculated. *P* values of ≤0.05 were considered significant.

### Ethics statement.

All studies were approved by the local ethics committee Comité de Protection des Personnes Ouest IV-Nantes and the Agence française de sécurité sanitaire des produits de santé. Written consent was obtained from all patients and healthy subjects.

## Supplementary Material

Supplemental material
