# Supplementary material for: Specific Human and Candida Cellular Interactions Lead to Controlled or Persistent Infection Outcomes during Granuloma-Like Formation
Source: Infect Immun. 2016 Dec 29;85(1):e00807-16. doi: 10.1128/IAI.00807-16 (PMC5203659; doi:10.1128/IAI.00807-16)
Supplement: Supplemental material [file supp_85_1_e00807-16__index.html]

Supplemental material 

# Specific Human and Candida Cellular Interactions Lead to Controlled or Persistent Infection Outcomes during Granuloma-Like Formation

## Supplemental material

- Supplemental file 1 -

  Fig. S1. Evolution profiles of the fungal burden within granulomatous structures of *Candida* spp. Fig. S2. Evolution of the fungal burden within granulomatous structures of 32 clinical isolates of *Candida* spp. Fig. S3. Evolution of the fungal burden within granulomatous structures of 32 clinical isolates of *Candida* spp. Table S1. Distribution of granulocyte, monocyte, and T cell subsets based on CD66, CD14, CD56, CD4, and CD8 from persistent-infection and controlled-infection granulomas after infection with *C. albicans*, *C. dubliniensis*, and *C. tropicalis* (group A). Table S2. Distribution of granulocyte, monocyte, and T cell subsets based on CD66, CD14, CD56, CD4, and CD8 from persistent-infection and controlled-infection granulomas after infection with *C. lusitaniae*, *C. parapsilosis*, and *C. glabrata* (group B). Table S3. Distribution of granulocyte, monocyte, and T cell subsets based on CD66, CD14, CD56, CD4, and CD8 from persistent-infection and controlled-infection granulomas after infection with *C. krusei* and *C. kefyr* (group C).

  PDF, 1.6M
- Supplemental file 2 -

  Video S1. Representative movie from microscopy time-lapse series.

  MOV, 5.1M
- Supplemental file 3 -

  Video S2. Representative movie from microscopy time-lapse series showing human leucocyte infected by GFP-tagged *C. albicans* blastoconidia (MOI of 2,000∶1).

  MOV, 9.1M
- Supplemental file 4 -

  Video S1 and S2 legends.

  PDF, 57K
