# Supplementary material for: Specific Human and Candida Cellular Interactions Lead to Controlled or Persistent Infection Outcomes during Granuloma-Like Formation
Source: Infect Immun. 2016 Dec 29;85(1):e00807-16. doi: 10.1128/IAI.00807-16 (PMC5203659; doi:10.1128/IAI.00807-16)
Supplement: Supplemental material [file IAI.00807-16_zii999091935s1.pdf]

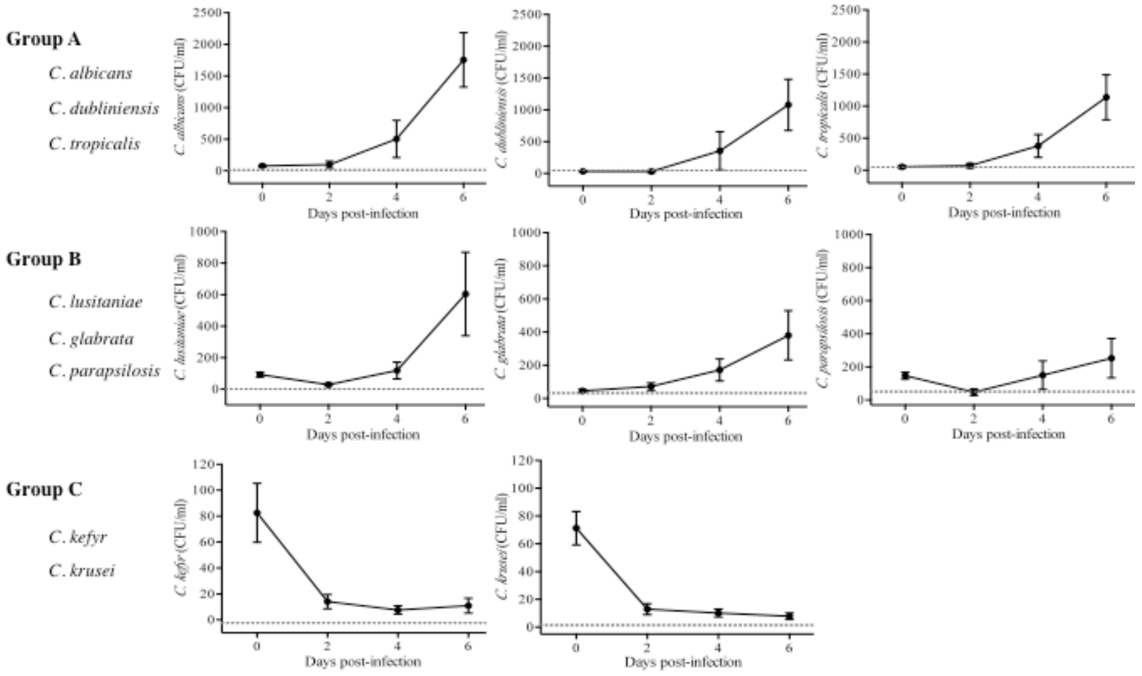

Fig. S1. Evolution profiles of the fungal burden within granulomatous structures of *Candida* spp. The fungal burden was followed at 0, 2, 4 and 6 days after infection of human peripheral blood mononuclear and polymorphonuclear cells with living yeasts from different *Candida* species. Results are expressed as colony-forming units per ml (CFU/ml). Data are represented as the mean + SEM of the fungal burden from ten healthy donors for each *Candida* species. Profiling was performed according to the two-way ANOVA statistical analysis ($\alpha = 0.05$). Three profiles were identified: profile A: *C. albicans*, *C. dubliniensis* and *C. tropicalis* showing “high” fungal burdens. Profile B: *C. lusitaniae*, *C. glabrata* and *C. parapsilosis* showing “intermediate” fungal burdens. Profile C: *C. kefyr* and *C. krusei* presenting “low” fungal burdens.

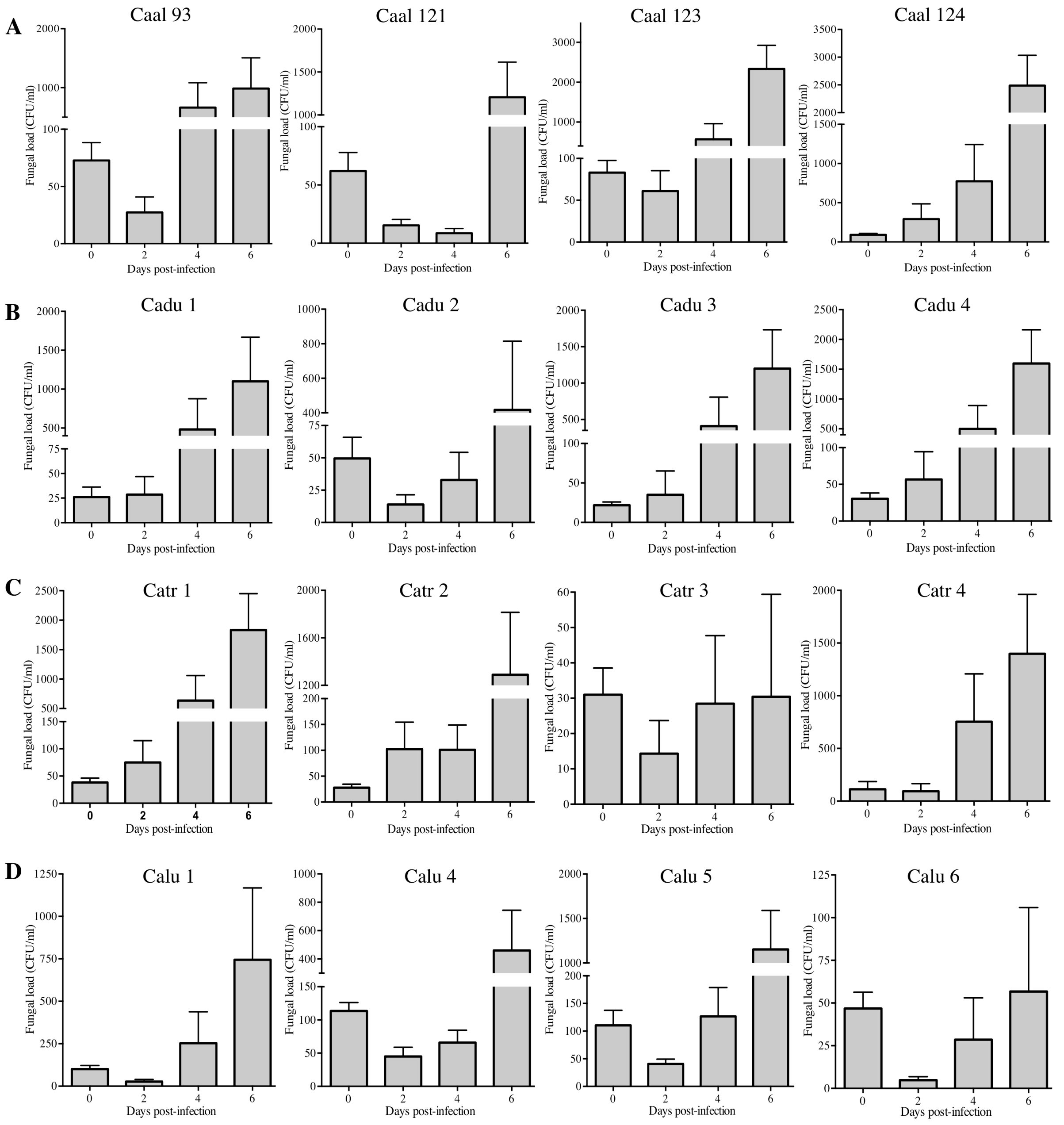

Fig. S2. Evolution of the fungal burden within granulomatous structures of 32 clinical isolates of *Candida* spp. The fungal burden was followed at 0, 2, 4 and 6 days after infection of human peripheral blood mononuclear and polymorphonuclear cells with living yeasts from different *Candida* species. Results are expressed as colony-forming units per ml (CFU/ml). Data are represented as the mean + SEM of the fungal burden from ten healthy donors for each *Candida* species: **(A)** *C. albicans*, **(C)** *C. dubliniensis*, **(C)** *C. tropicalis* and **(D)** *C. lusitaniae*.

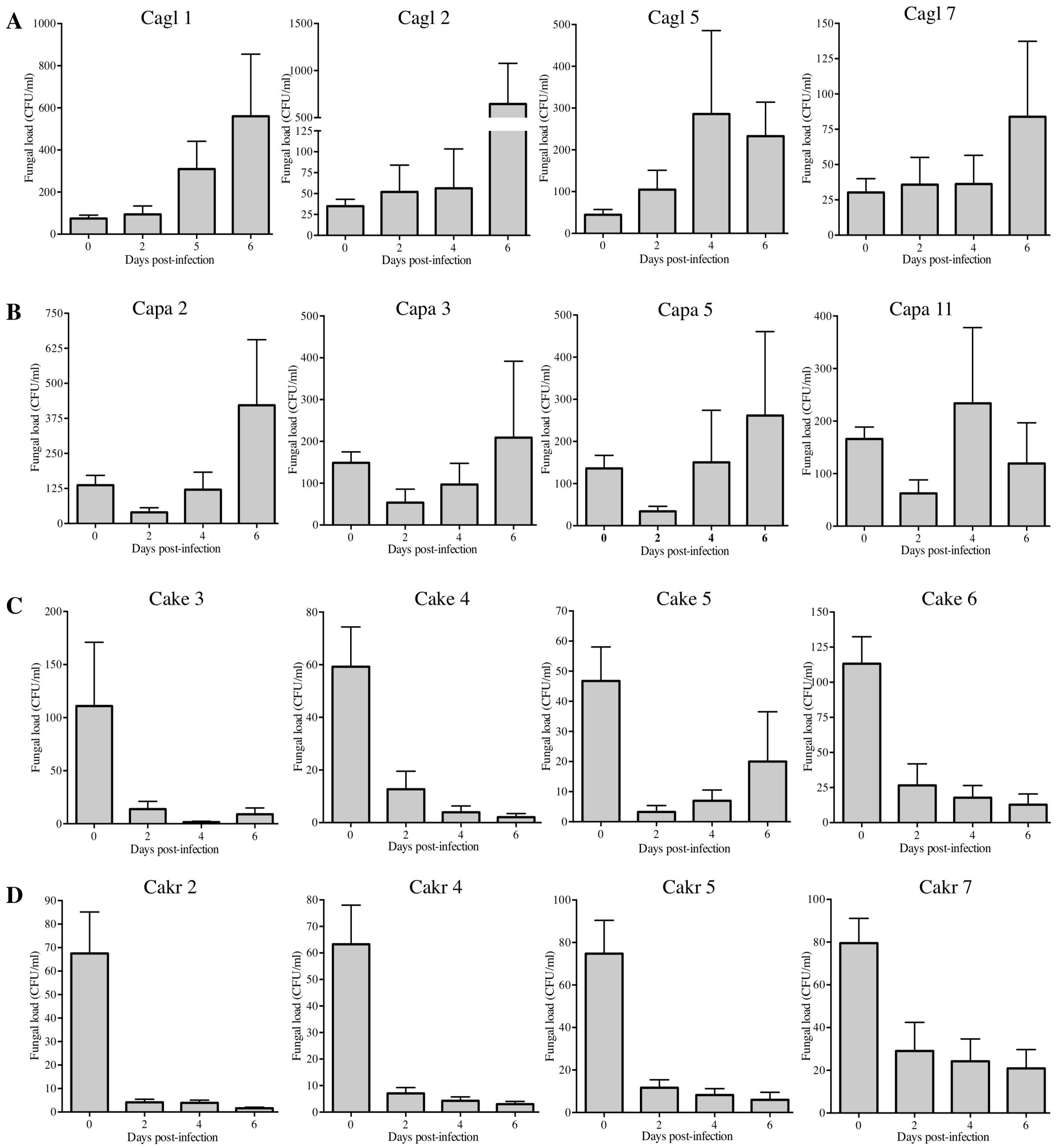

Fig. S3. Evolution of the fungal burden within granulomatous structures of 32 clinical isolates of *Candida* spp. The fungal burden was followed at 0, 2, 4 and 6 days after infection of human peripheral blood mononuclear and polymorphonuclear cells with living yeasts from different *Candida* species. Results are expressed as colony-forming units per ml (CFU/ml). Data are represented as the mean + SEM of fungal burden from ten healthy donors for each *Candida* species: **(A)** *C. glabrata*, **(B)** *C. parapsilosis*, **(C)** *C. kefyr* and **(D)** *C. krusei*.

Table S1. Distribution of granulocyte, monocyte and T cell subsets based on CD66, CD14, CD56, CD4 and CD8 from persistent-infection and controlled-infection granulomas after infection with *C. albicans*, *C. dubliniensis* and *C. tropicalis* (group A).

	**Day 0**				**Day 2**				**Day 4**				**Day 6**			
	**Mean (SD) PI**	**Mean (SD) CI**	***p***	**Mean Diff.**	**Mean (SD) PI**	**Mean (SD) CI**	***p***	**Mean Diff.**	**Mean (SD) PI**	**Mean (SD) CI**	***p***	**Mean Diff.**	**Mean (SD) PI**	**Mean (SD) CI**	***p***	**Mean Diff.**
**CD66+**	40.24 (9.95)	60.33 (13.96)	****	-20.1	26.26 (18.64)	46.78 (13.31)	****	-20.5	14.57 (6.32)	15.95 (5.206)	ns	-1.3	7.15 (7.77)	12.22 (6.41)	ns	-5.1
**CD14+**	6.31 (2.35)	5.94 (0.936)	ns	0.4	5.43 (3.767)	7.133 (1.94)	ns	-1.7	4.60 (4.51)	6.14 (3.11)	ns	-2	1.16 (1.41)	3.52 (2.55)	*	-2.4
**CD3-CD56+**	14.26 (4.93)	12.13 (4.864)	ns	2.2	15.2 (6.82)	12.96 (5.12)	ns	2.3	11.5 (4.952)	8.30 (3.969)	ns	3.2	13.55 (8.40)	4.81 (3.033)	****	8.7
**CD3+CD56+**	5.56 (6.13)	4.28 (1.27)	ns	1.2	4.01 (3.30)	2.923 (0.92)	ns	1.1	4.45 (4.44)	2.48 (1.348)	ns	2	4.53 (5.43)	2.36 (1.10)	ns	2.2
**CD3+**	60.49 (9.42)	68.53 (3.435)	ns	3.1	50.37 (16.17)	59.70 (7.75)	*	-9.1	61.55 (13.36)	63.51 (12.71)	ns	-1.9	50.92 (12.29)	65.88 (17.77)	****	-15
**CD4+CD8-**	45.85 (11.67)	58.85 (3.993)	****	-13	40.07 (11.96)	51.78 (6.931)	***	-11.7	45.65 (14.76)	58.31 (10.78)	****	-12.7	41.46 (12.16)	59.76 (15.08)	****	-18.3
**CD4-CD8+**	26.19 (8.76)	18.96 (3.263)	ns	4.8	23.83 (8.84)	15.67 (2.412)	***	8.1	23.46 (9.395)	10.59 (3.33)	****	12.9	20.15 (9.844)	11.29 (3.68)1	****	8.9
**CD4+:CD8+ ratio**	1.75	3.1			1.68	3.3			1.94	5.51			2.06	5.29		
**CD4hiCD8hi**	0.20 (0.17)	0.22 (0.04)	ns	0	0.33 (0.43)	0.15 (0.12)	ns	0.2	0.36 (0.50)	0.15 (0.11)	*	0.4	0.13 (0.17)	0.07 (0.06)	ns	0.1
**CD4hiCD8lo**	0.57 (0.55)	0.55 (0.30)	ns	0	0.47 (0.6434)	0.38 (0.30)	ns	0.1	0.59 (0.85)	0.35 (0.23)	ns	0.2	0.67 (0.80)	0.37 (0.36)	ns	0.3
**CD4loCD8hi**	0.93 (0.76)	0.47 (0.22)	ns	0.5	0.73 (0.89)	0.31 (0.18)	ns	0.4	1.02 (1.441)	0.23 (0.15)	**	0.8	1.32 (1.079)	0.46 (0.31)	***	0.9

Results were expressed as the median fraction of cells expressing the marker(s) +/- SD among specific gates. Significant differences were evaluated by comparison between persistent-infection (PI) and controlled-infection (CI) status over time using one-way ANOVA with Tukey's multiple comparisons test.*, $p < 0.05$; **, $p < 0.001$; ***, $p < 0.0001$; ****, $p < 0.00001$, ns: not significant.

Table S2. Distribution of granulocyte, monocyte and T cell subsets based on CD66, CD14, CD56, CD4 and CD8 from persistent-infection and controlled-infection granulomas after infection with *C. lusitaniae*, *C. parapsilosis* and *C. glabrata* (group B).

	**Day 0**				**Day 2**				**Day 4**				**Day 6**			
	**Mean (SD) PI**	**Mean (SD) CI**	***p***	**Mean Diff.**	**Mean (SD) PI**	**Mean (SD) CI**	***p***	**Mean Diff.**	**Mean (SD) PI**	**Mean (SD) CI**	***p***	**Mean Diff.**	**Mean (SD) PI**	**Mean (SD) CI**	***p***	**Mean Diff.**
**CD66+**	41.25 (12.93)	53.87 (14.91)	****	-12.6	31.33 (18.40)	42.06 (16.82)	****	-22.5	15.96 (4.730)	16.67 (4.73)	ns	-0.7	6.73 (6.82)	9.48 (7.70)	ns	-2.7
**CD14+**	6.11 (2.075)	6.579 (1.054)	ns	-0.3	6.10 (3.70)	6.48 (3.22)	ns	-1.8	3.88 (2.77)	5.71 (3.76)	ns	-1.1	2.70 (2.61)	2.53 (2.81)	ns	-0.1
**CD3-CD56+**	13.98 (4.79)	13.12 (5.19)	ns	0.8	15.32 (6.80)	13.37 (6.50)	ns	10.5	13.32 (6.03)	9.11 (4.42)	**	4.2	9.86 (5.404)	5.95 (3.53)	*	3.9
**CD3+CD56+**	5.91 (5.733)	4.618 (4.402)	ns	1.2	4.35 (3.317)	3.03 (2.33)	ns	1.3	4.14 (3.88)	3.50 (3.01)	ns	0.6	4.27 (4.98)	3.38 (4.67)	ns	0.8
**CD3+**	62.31 (10.05)	64.03 (7.316)	ns	-1.7	52.68 (13.42)	52.32 (13.01)	ns	0.3	59.47 (11.41)	65.06 (11.33)	ns	-5.5	53.30 (13.61)	56.87 (17.85)	ns	-3.5
**CD4+CD8-**	48.30 (12.44)	52.06 (10.36)	ns	-3.7	41.24 (12.71)	43.66 (10.93)	ns	-2.4	46.34 (13.71)	53.64 (14.49)	ns	-7.3	43.31 (14.01)	47.93 (18.15)	ns	-4.6
**CD4-CD8+**	26.21 (8.43)	21.35 (7.107)	ns	4.8	24.75 (9.74)	17.84 (8.23)	**	6.9	22.19 (10.52)	16.65 (8.96)	ns	5.6	19.15 (8.60)	14.83 (9.287)	ns	4.3
**CD4+:CD8+ ratio**	1.84	2.44			1.67	2.45			2.08	3.22			2.26	3.23		
**CD4hiCD8hi**	0.20 (0.13)	0.23 (0.16)	ns	0	0.28 (0.36)	0.26 (0.43)	ns	0	0.28 (0.34)	0.33 (0.45)	ns	-0.1	0.11 (0.12)	0.11 (0.13)	ns	0
**CD4hiCD8lo**	0.44 (0.32)	0.52 (0.36)	ns	-0.1	0.32 (0.29)	0.39 (0.35)	ns	-0.1	0.36 (0.29)	0.40 (0.32)	ns	0	0.45 (0.36)	0.43 (0.45)	ns	0
**CD4loCD8hi**	0.70(0.58)	0.88 (0.78)	ns	-0.2	0.62 (0.75)	0.66 (0.10)	ns	0	0.72 (0.91)	0.83 (1.31)	ns	-0.1	0.94 (0.82)	0.10 (1.14)	ns	0

Results were expressed as the median fraction of cells expressing the marker(s) +/- SD among specific gates. Significant differences were evaluated by comparison between persistent-infection (PI) and controlled-infection (CI) status over time using one-way ANOVA with Tukey's multiple comparisons test.*, $p < 0.05$; **, $p < 0.001$; ***, $p < 0.0001$; ****, $p < 0.00001$, ns: not significant.

Table S3. Distribution of granulocyte, monocyte and T cell subsets based on CD66, CD14, CD56, CD4 and CD8 from persistent-infection and controlled-infection granulomas after infection with *C. krusei* and *C. kefyr* (group C).

	**Day 0**			**Day 2**			**Day 4**			**Day 6**		
	**Mean (SD) CI**	***p* vs. group A CI**	***p* vs. group B CI**	**Mean (SD) IC**	***p* vs. group A CI**	***p* vs. group B CI**	**Mean (SD) CI**	***p* vs. group A CI**	***p* vs. group B CI**	**Mean (SD) CI**	***p* vs. group A CI**	***p* vs. group B CI**
**CD66+**	48.61 (15.42)	***	ns	36.35 (18.29)	**	ns	16.13 (5.17)	ns	ns	8.50 (7.41)	ns	ns
**CD14+**	5.9 (2.172)	ns	ns	6.50 (3.41)	ns	ns	4.94 (4.03)	ns	ns	2.48 (2.22)	ns	ns
**CD3-CD56+**	13.55 (5.00)	ns	ns	15.98 (7.10)	*	ns	10.28 (4.58)	ns	ns	7.79 (4.32)	**	ns
**CD3+CD56+**	5.14 (5.10)	ns	ns	3.619 (2.90)	ns	ns	3.74 (3.62)	ns	ns	3.79 (4.71)	ns	ns
**CD3+**	63.17 (8.81)	ns	ns	52.58 (13.12)	ns	ns	63.05 (11.30)	ns	ns	56.01 (14.63)	**	ns
**CD4+CD8-**	50.18 (11.58)	ns	ns	42.79 (11.84)	ns	ns	50.76 (14.04)	ns	ns	46.16 (15.00)	****	ns
**CD4-CD8+**	23.78 (8.15)	ns	ns	22.24 (9.20)	*	ns	18.84 (9.82)	***	ns	17.65 (9.46)	*	ns
**CD4+:CD8+ ratio**	2.11			1.92			2.69			2.61		
**CD4hiCD8hi**	0.21 (0.15)	ns	ns	0.28 (0.37)	ns	ns	0.29 (0.43)	ns	ns	0.11 (0.12)	ns	ns
**CD4hiCD8lo**	0.49 (0.35)	ns	ns	0.34 (0.35)	ns	ns	0.40 (0.34)	ns	ns	0.51 (0.49)	ns	ns
**CD4loCD8hi**	0.79 0.69)	ns	ns	0.65 (0.888)	ns	ns	0.79 (1.17)	ns	ns	1.00 (1.00)	ns	ns

Results are expressed as median fraction of cells expressing the marker(s) +/- SD among specific gates. Significant differences were evaluated by comparison of controlled-infection (CI) status between profile A and B over time using one-way ANOVA with Tukey's multiple comparisons test.*, $p < 0.05$; **, $p < 0.001$; ***, $p < 0.0001$; ****, $p < 0.00001$, ns: not significant.
