# Supplementary material for: Specific Human and Candida Cellular Interactions Lead to Controlled or Persistent Infection Outcomes during Granuloma-Like Formation
Source: Infect Immun. 2016 Dec 29;85(1):e00807-16. doi: 10.1128/IAI.00807-16 (PMC5203659; doi:10.1128/IAI.00807-16)
Supplement: Supplemental material [file IAI.00807-16_zii999091935s4.pdf]

Video S1. Representative movie from microscopy time lapse series. Human leucocytes were infected with GFP-tagged *C. albicans* blastoconidia at a MOI of 2000:1. Phagocytosis and host response against *C. albicans* filaments was detected over 72 h of incubation with a 300 W xenon lamp fitted with a 488 nm excitation filter. Emission at 515 nm was used for analysis of *C. albicans* fluorescence with a Leica DMI6000B camera cool Snap HQ2 and processed with Metamorph imaging software version 7.7.4.0.

Video S2. Representative movie from microscopy time lapse series showing humain leucocyte infected by GFP-tagged *C. albicans* blastoconidia (MOI of 2000:1). Cells were illuminated at day 0 and every 10 min over 72 h of incubation with a 300 W xenon lamp fitted with a 488 nm excitation filter. Emission at 515 nm was used for analysis of *C. albicans* fluorescence with a Leica DMI6000B camera cool Snap HQ2 and processed with Metamorph imaging software version 7.7.4.0.
